# Increases in ambient air pollutants during pregnancy are linked to increases in methylation of IL4, IL10, and IFNγ

**DOI:** 10.1186/s13148-022-01254-2

**Published:** 2022-03-14

**Authors:** Juan Aguilera, Xiaorui Han, Shu Cao, John Balmes, Fred Lurmann, Tim Tyner, Liza Lutzker, Elizabeth Noth, S. Katharine Hammond, Vanitha Sampath, Trevor Burt, P. J. Utz, Purvesh Khatri, Nima Aghaeepour, Holden Maecker, Mary Prunicki, Kari Nadeau

**Affiliations:** 1 Sean N. Parker Center for Allergy and Asthma Research, Stanford University, 240 Pasteur, Stanford, CA 94305 USA; 2 Division of Environmental Health Sciences, School of Public Health, University of California, Berkeley, CA USA; 3 Department of Medicine, University of California, San Francisco, San Francisco, CA USA; 4 Sonoma Technology, Petaluma, CA USA; 5 University of California, San Francisco-Fresno, Fresno, CA USA; 6 Central California Asthma Collaborative, Fresno, USA; 7 Department of Pediatrics, Division of Neonatology and the Translating Duke Health Children’s Health and Discovery Initiative, Duke University School of Medicine, 701 W Main St., Chesterfield Building, Suite 510, Durham, NC 27701 USA; 8 Department of Medicine, Division of Immunology and Rheumatology, Stanford University School of Medicine, Stanford University, 291 Campus Drive, Stanford, CA 94305 USA; 9 Center for Biomedical Informatics, Department of Medicine, Stanford University School of Medicine, Stanford University, 291 Campus Drive, Stanford, CA 94305 USA; 10 Departments of Biomedical Data Sciences, Stanford University, 291 Campus Drive, Stanford, CA 94305 USA; 11 Institute for Immunity, Transplantation and Infection, Stanford University, 291 Campus Drive, Stanford, CA 94305 USA

**Keywords:** Air pollution, DNA methylation, Pregnancy, Immunology, Th1, Th2, IL4, IL10, IFNγ, PM_2.5_

## Abstract

**Background:**

Ambient air pollutant (AAP) exposure is associated with adverse pregnancy outcomes, such as preeclampsia, preterm labor, and low birth weight. Previous studies have shown methylation of immune genes associate with exposure to air pollutants in pregnant women, but the cell-mediated response in the context of typical pregnancy cell alterations has not been investigated. Pregnancy causes attenuation in cell-mediated immunity with alterations in the Th1/Th2/Th17/Treg environment, contributing to maternal susceptibility. We recruited women (*n* = 186) who were 20 weeks pregnant from Fresno, CA, an area with chronically elevated AAP levels. Associations of average pollution concentration estimates for 1 week, 1 month, 3 months, and 6 months prior to blood draw were associated with Th cell subset (Th1, Th2, Th17, and Treg) percentages and methylation of CpG sites (*IL4*, *IL10, IFNγ,* and *FoxP3*). Linear regression models were adjusted for weight, age, season, race, and asthma, using a *Q* value as the false-discovery-rate-adjusted *p*-value across all genes.

**Results:**

Short-term and mid-term AAP exposures to fine particulate matter (PM_2.5_), nitrogen dioxide (NO_2_) carbon monoxide (CO), and polycyclic aromatic hydrocarbons (PAH_456_) were associated with percentages of immune cells. A decrease in Th1 cell percentage was negatively associated with PM_2.5_ (1 mo/3 mo: *Q* < 0.05), NO_2_ (1 mo/3 mo/6 mo: *Q* < 0.05), and PAH_456_ (1 week/1 mo/3 mo: *Q* < 0.05). Th2 cell percentages were negatively associated with PM_2.5_ (1 week/1 mo/3 mo/6 mo: *Q* < 0.06), and NO_2_ (1 week/1 mo/3 mo/6 mo: *Q* < 0.06). Th17 cell percentage was negatively associated with NO_2_ (3 mo/6 mo: *Q* < 0.01), CO (1 week/1 mo: *Q* < 0.1), PM_2.5_ (3 mo/6 mo: *Q* < 0.05), and PAH_456_ (1 mo/3 mo/6 mo: *Q* < 0.08). Methylation of the *IL10* gene was positively associated with CO (1 week/1 mo/3 mo: *Q* < 0.01), NO_2_ (1 mo/3 mo/6 mo: *Q* < 0.08), PAH_456_ (1 week/1 mo/3 mo: *Q* < 0.01), and PM_2.5_ (3 mo: *Q* = 0.06) while *IL4* gene methylation was positively associated with concentrations of CO (1 week/1 mo/3 mo/6 mo: *Q* < 0.09). Also, *IFNγ* gene methylation was positively associated with CO (1 week/1 mo/3 mo: *Q* < 0.05) and PAH_456_ (1 week/1 mo/3 mo: *Q* < 0.06).

**Conclusion:**

Exposure to several AAPs was negatively associated with T-helper subsets involved in pro-inflammatory and anti-inflammatory responses during pregnancy. Methylation of *IL4, IL10*, and *IFNγ genes* with pollution exposure confirms previous research. These results offer insights into the detrimental effects of air pollution during pregnancy, the demand for more epigenetic studies, and mitigation strategies to decrease pollution exposure during pregnancy.

**Supplementary Information:**

The online version contains supplementary material available at 10.1186/s13148-022-01254-2.

## Background

Ambient air pollutant (AAP) exposure is associated with adverse pregnancy outcomes, such as preeclampsia, preterm labor, and low birth weight, but the immune mechanisms involved are not clearly understood [1–4]. T-helper (Th) cells modulate immune responses and can be broadly classified into Th1 cells, involved in cellular immunity, and Th2 cells, involved in humoral immunity. In a typical pregnancy, there is an attenuation in cell-mediated immunity which shifts from a Th1 to a Th2-dominant environment [5, 6], contributing to overall maternal susceptibility to intracellular pathogens [7, 8] and air pollutants [9]; this environment of immune tolerance seems to dominate in the second trimester [10].

Immune alterations in utero may occur due to environmental factors such as smoking [11, 12], exposure to secondhand smoke [13], and elevated exposure to AAPs [14, 15]. AAP exposure can also lead to cytokine dysregulation and T cell polarization associated with epigenetic modifications [16]. These environmental impacts during pregnancy highlight the need to better understand immune alterations from AAP exposure and find effective ways to mitigate, treat, and/or prevent any adverse health effects to both mother and baby.

More specifically, Th1 cells produce interferon-gamma (IFNγ) which is considered pro-inflammatory while Th2 cells, produce the anti-inflammatory interleukins (IL)4 and IL10 [17, 18]. During pregnancy, Th2 cytokines promote placental changes, differentiation, and inhibition of pro-inflammatory Th1 cytokines [19, 20]. However, the Th1/Th2 paradigm has more recently expanded into a Th1/Th2/Th17 and T-regulatory (Treg) paradigm [18, 21]. Th17 cells cause inflammation which may play an important role in preventing pathological infections during pregnancy but have also been associated with preeclampsia [21]. In contrast, Treg cells, which are identified by the expression of FoxP3, play a central role in immunoregulation and tolerance, including maternal–fetal tolerance during pregnancy [18].

Studies have linked decreases in levels of IL10 with exposure to AAPs such as nitrogen oxides (NOx) [22] and PM_10_ [23]. Others have found that exposure to particulate matter leads to the elevation of IL4 in animal studies [24]. Our group previously showed that exposure to polycyclic aromatic hydrocarbons with 4,5,6-rings (PAH_456_) is associated with decreased *IL10* gene methylation [25]. Furthermore, we have found links between AAPs and DNA methylation of specific CpG sites of *IL10* and *FoxP3* genes in children with asthma [26] and more recently that AAP exposure was associated with altered methylation of CpG sites for *IFNγ, IL4, IL10*, and *FoxP3* genes [27].

Residents from Fresno, California, have consistently elevated exposure to AAPs. According to the 2020 *State Of The Air* report for US cities, Fresno is ranked first in short-term particle pollution, second in year-round particle pollution, and fourth in ozone (O_3_) exposure [28]. Here, we identified differentially methylated regions in the maternal *IFNγ, IL4, IL10,* and *FoxP3* genes during pregnancy and associated them with exposure to AAPs. Our primary hypothesis is that exposures to elevated levels of elemental carbon (EC), CO, O_3_, NO_2_, NOx, PAH_456_, PM_2.5_, and coarse particulate matter (PM_10_) are associated with increased methylation of the *IL4, IL10, IFNγ*, and *FoxP3* genes. Our secondary hypothesis is that Th cell percentages are associated with exposure to high levels of AAPs. In addition, based on our previous findings of exposure to AAPs and gene methylation in children [26, 27], we aimed to explore associations between altered methylation of *IFNγ, IL4, IL10*, and *FoxP3* genes from the mothers and their children’s cord blood, as well as blood samples drawn in these offspring at 12 and 24 months old.

## Results

### Subject characteristics

The demographics of the 186 women recruited at aproximately 20 weeks of pregnancy are summarized in Table 1. The median age (quartile 1; quartile 3) was 27 years (23; 33). The median weight at the start of the pregnancy (quartile 1; quartile 3) was 72.73 kg (59.09; 85.00). Most enrolled subjects were Hispanic (73%).

**Table 1:** Demographic characteristics of subjects

	Overall
	*N* = 186
Age of mother at baseline (median [IQR]) years	27 (23, 33)
Weight at start of pregnancy (kgs) (median [IQR])	72.7 [59.1, 85.0]
Race n (%)	
Hispanic	135 (72.6)
African American	20 (10.8)
White	17 (9.1)
Asian or Pacific Islander	13 (7.0)
Household income last year before taxes (%)	
≤ $15 K	59 (31.7)
> $15–$30 K	78 (41.9)
> $30–$50 K	31 (16.7)
> $50 K	6 (3.2)
Number of weeks pregnant (median [IQR])	22 (20, 23)
Season of baseline visit (%)	
1: Oct–Jan (winter)	54 (29.0)
2: Feb–May (spring)	64 (34.4)
3: Jun–Sep (summer)	68 (36.6)
Ever diagnosed Asthma (%)	44 (23.7)

The mean AAP exposure averages were calculated at 1 week 1, 3, and 6 months before each subject’s blood draw (Additional file 1: Fig. S1).

### Methylation is associated with ambient air pollution

We explored associations between AAP exposures (CO, elemental carbon (EC), O_3_, NO_2_, NOx, PAH_456_, PM_10_, PM_2.5_) and methylation of CpG sites in the *IFNγ, IL4, IL10,* and *FoxP3* genes. We constructed a linear regression model on each individual gene methylation as a function of each AAP exposure, adjusting for weight, age, season, race, and asthma. The *p*-values for the association with exposure across all genes were adjusted for multiple testing using the Benjamini–Hochberg procedures that control the false-discovery rate (FDR). We used a *Q* value which denotes the adjusted *p*-value across all genes based on the regression model, and *Q* < 0.1 was considered as statistically significant. Coefficients and corresponding 95% confidence intervals for each AAP exposure were summarized in Additional file 19: Table S3.

**Fig. 1:**
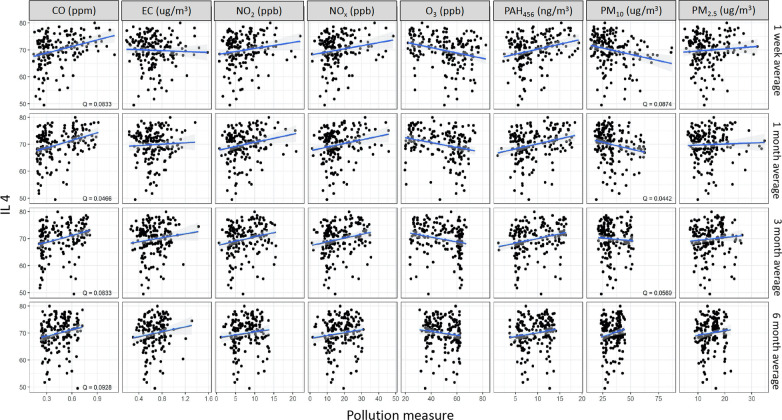
Associations between IL4 and ambient air pollutant levels. *Q* value is the false-discovery-rate-adjusted *p*-value across all genes, based on linear regression model adjusting for weight, age, season, race, and asthma diagnosis. *Q* < 0.1 is considered statistically significant. IL4 refers to average DNA methylation across 6 CpG sites in the gene. CO: Carbon monoxide, EC: Elemental carbon, NO_2_: Nitric dioxide, NOx: Nitric oxides, O_3_: Ozone, PAH: Polycyclic aromatic hydrocarbons, PM: particulate matter

**Fig. 2:**
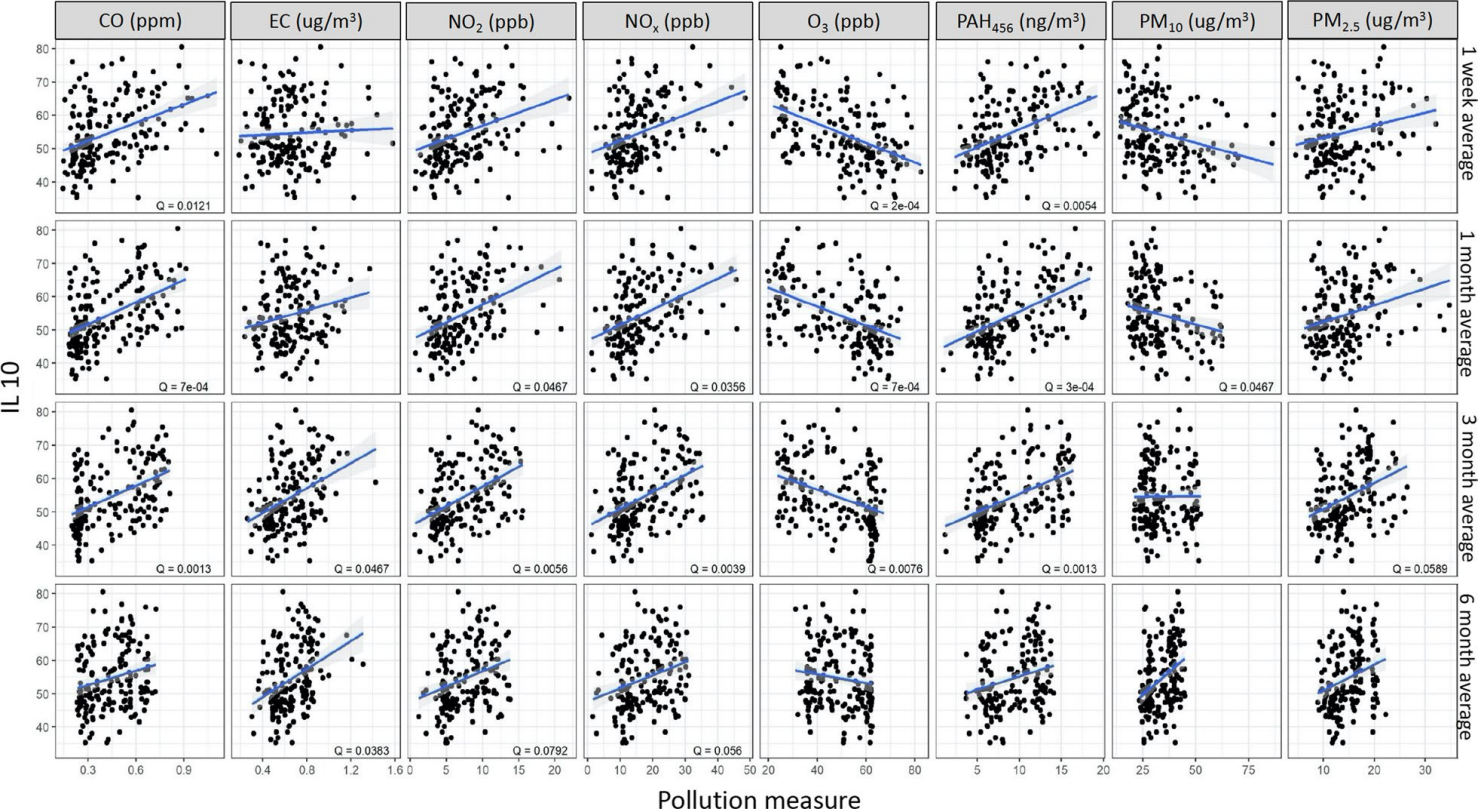
Associations between IL10 and ambient air pollutant levels. *Q* value is the false-discovery-rate-adjusted *p*-value across all genes, based on linear regression model adjusting for weight, age, season, race, and asthma diagnosis. *Q* < 0.1 is considered statistically significant. IL10 refers to average DNA methylation across 4 CpG sites in the gene. CO: Carbon monoxide, EC: Elemental carbon, NO_2_: Nitric dioxide, NOx: Nitric oxides, O_3_: Ozone, PAH: Polycyclic aromatic hydrocarbons, PM: particulate matter

**Fig. 3:**
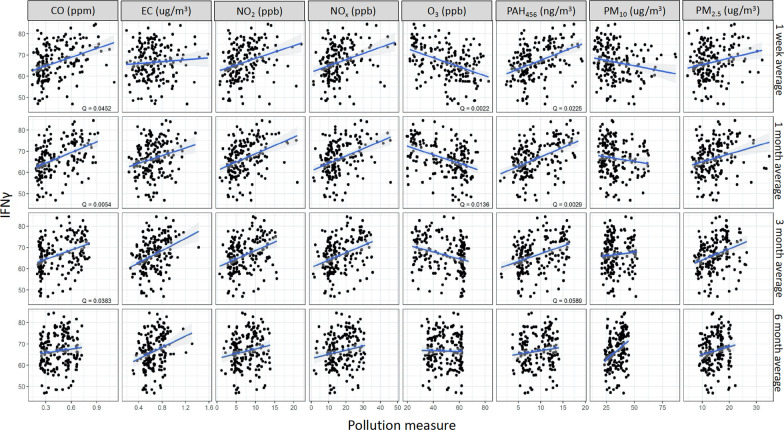
Associations between IFNγ and ambient air pollutant levels. *Q* value is the false-discovery-rate-adjusted *p*-value across all genes, based on linear regression model adjusting for weight, age, season, race, and asthma diagnosis. *Q* < 0.1 is considered statistically significant. IFNγ refers to average DNA methylation across 3 CpG sites in the gene. CO: Carbon monoxide, EC: Elemental carbon, NO_2_: Nitric dioxide, NOx: Nitric oxides, O_3_: Ozone, PAH: Polycyclic aromatic hydrocarbons, PM: particulate matter

Methylation of *IL4* was positively associated with CO (1 w: *Q* = 0.08, 1 m: *Q* = 0.05, 3 m: *Q* = 0.08 and 6 m: *Q* = 0.09) and negatively associated with PM_10_ (1 w: *Q* = 0.08, 1 m: Q = 0 0.04, 3 m: *Q* = 0.06) (Fig. 1**)**. Methylation of *IL10* was positively associated with CO (1 w: *Q* = 0.01, 1 m: *Q* < 0.01, 3 m: *Q* < 0.01), EC (3 m: *Q* = 0.05 and 6 m: *Q* = 0.04), NO_2_ (1 m: *Q* = 0.05, 3 m: *Q* < 0.01 and 6 m: *Q* = 0.08), NOx (1 m: *Q* = 0.04, 3 m: *Q* < 0.01 and 6 m: *Q* = 0.06), PAH_456_ (1 w: *Q* < 0.01, 1 m: *Q* < 0.01 and 3 m: *Q* < 0.01), PM_2.5_ (3 m: *Q* = 0.06) and negatively associated with O_3_ (1 w: *Q* < 0.01, 1 m: *Q* < 0.01 and 3 m: *Q* < 0.01) and PM_10_ (1 m: *Q* = 0.05) (Fig. 2). In addition, *IFNγ* methylation was positively associated with CO (1 w: *Q* = 0.05, 1 m: *Q* = 0.01, and 3 m: *Q* = 0.04) and PAH_456_ (1 w: *Q* = 0.02, 1 m: *Q* < 0.01, 3 m: *Q* = 0.06) (Fig. 3) while *FoxP3* methylation was only positively associated with PM_10_ (6 m: *Q* = 0.05) (Additional file 2: Fig. S2).

### Frequency of cell populations is associated with ambient air pollution

**Fig. 4:**
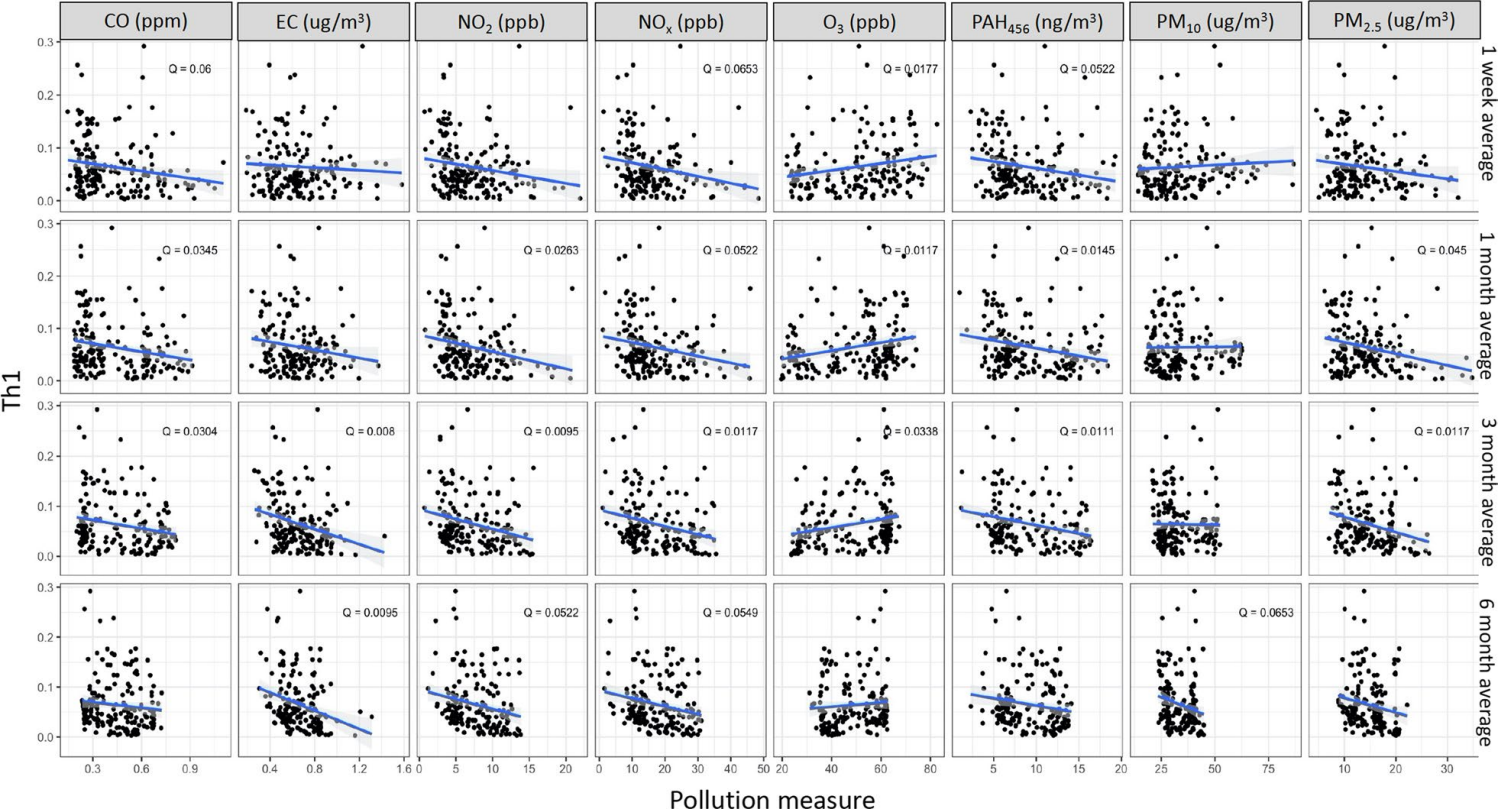
Associations between Th1 percentage and Ambient Air Pollutant levels. *Q* value is the false-discovery-rate-adjusted *p*-value across all genes, based on linear regression model adjusting for weight, age, season, race, and asthma diagnosis. *Q* < 0.1 is considered statistically significant. CO: Carbon monoxide, EC: Elemental carbon, NO_2_: Nitric dioxide, NOx: Nitric oxides, O_3_: Ozone, PAH: Polycyclic aromatic hydrocarbons, PM: particulate matter

**Fig. 5:**
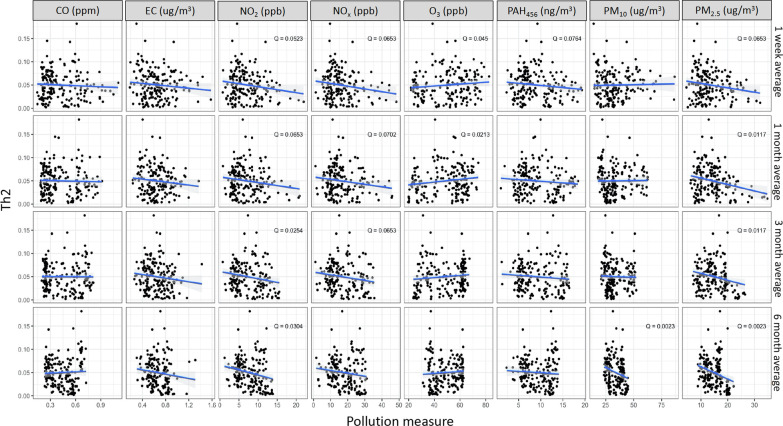
Associations between Th2 percentage and ambient air pollutant levels. *Q* value is the false-discovery-rate-adjusted *p*-value across all genes, based on linear regression model adjusting for weight, age, season, race, and asthma diagnosis. *Q* < 0.1 is considered statistically significant. CO: Carbon monoxide, EC: Elemental carbon, NO_2_: Nitric dioxide, NOx: Nitric oxides, O_3_: Ozone, PAH: Polycyclic aromatic hydrocarbons, PM: particulate matter

**Fig. 6:**
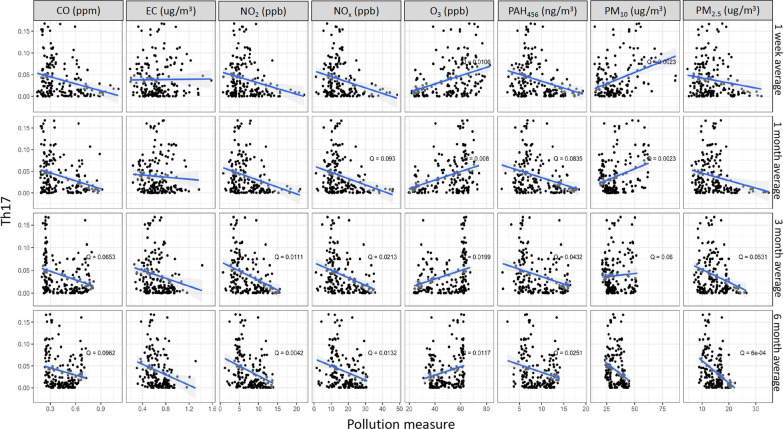
Associations between Th17 percentage and ambient air pollutant levels. *Q* value is the false-discovery-rate-adjusted *p*-value across all genes, based on linear regression model adjusting for weight, age, season, race, and asthma diagnosis. *Q* < 0.1 is considered statistically significant. CO: Carbon monoxide, EC: Elemental carbon, NO_2_: Nitric dioxide, NOx: Nitric oxides, O_3_: Ozone, PAH: Polycyclic aromatic hydrocarbons, PM: particulate matter

Figure 4 illustrates the percentage of Th1 cells negatively associated with exposures to PM_2.5_ (1 m: *Q* = 0.05 and 3 m: *Q* = 0.01), NO_2_ (1 m: *Q* = 0.03, 3 m: *Q* = 0.01, and 6 m: *Q* = 0.05), NO_x_ (1 w: *Q* = 0.07, 1 m: *Q* = 0.05, 3 m: *Q* = 0.01, 6 m: *Q* = 0.05), and PAH_456_ (1 w: *Q* = 0.05, 1 m: *Q* = 0.01, and 3 m: *Q* = 0.01). We present in Fig. 5 the percentages of Th2 cells which were negatively associated with exposures to NO_2_ (1 w: *Q* = 0.05, 1 m: *Q* = 0.06, 3 m: *Q* = 0.02 and 6 m: *Q* = 0.03), NO_x_ (1 w: *Q* = 0.07, 1 m: *Q* = 0.07, 3 m: *Q* = 0.07), PAH_456_ (1 w: *Q* = 0.08) and PM_2.5_ (1 w: *Q* = 0.06, 1 m: *Q* = 0.01, 3 m: *Q* = 0.01 and 6 m: *Q* < 0.01) and positively associated with exposure to O_3_ (1 w: *Q* = 0.04, 1 m: *Q* = 0.02). Lastly, Fig. 6 shows that Th17 was negatively associated with exposures to CO (3 w: *Q* = 0.07, 1 m: *Q* < 0.1), NO_2_ (3 w: *Q* = 0.01, 6 m: *Q* = 0.004), NO_x_ (1 m: *Q* < 0.1, 3 m: *Q* = 0.02, 6 m: *Q* = 0.01), PAH_456_ (1 m: *Q* = 0.8, 3 m: *Q* = 0.04, 6 m: *Q* = 0.03) and PM_2.5_ (3 m: *Q* = 0.05 and 6 m: *Q* < 0.01) and positively associated with exposures to O_3_ (1 m: *Q* < 0.01,1 3 m: *Q* = 0.04, 6 m: *Q* = 0.02) and PM_10_.(1 w: *Q* < 0.01, 1 m: *Q* < 0.01. 3 m: *Q* = 0.06). No associations were found between Treg cell percentages and AAP (Additional file 3: Fig.S3).

**Fig. 7:**
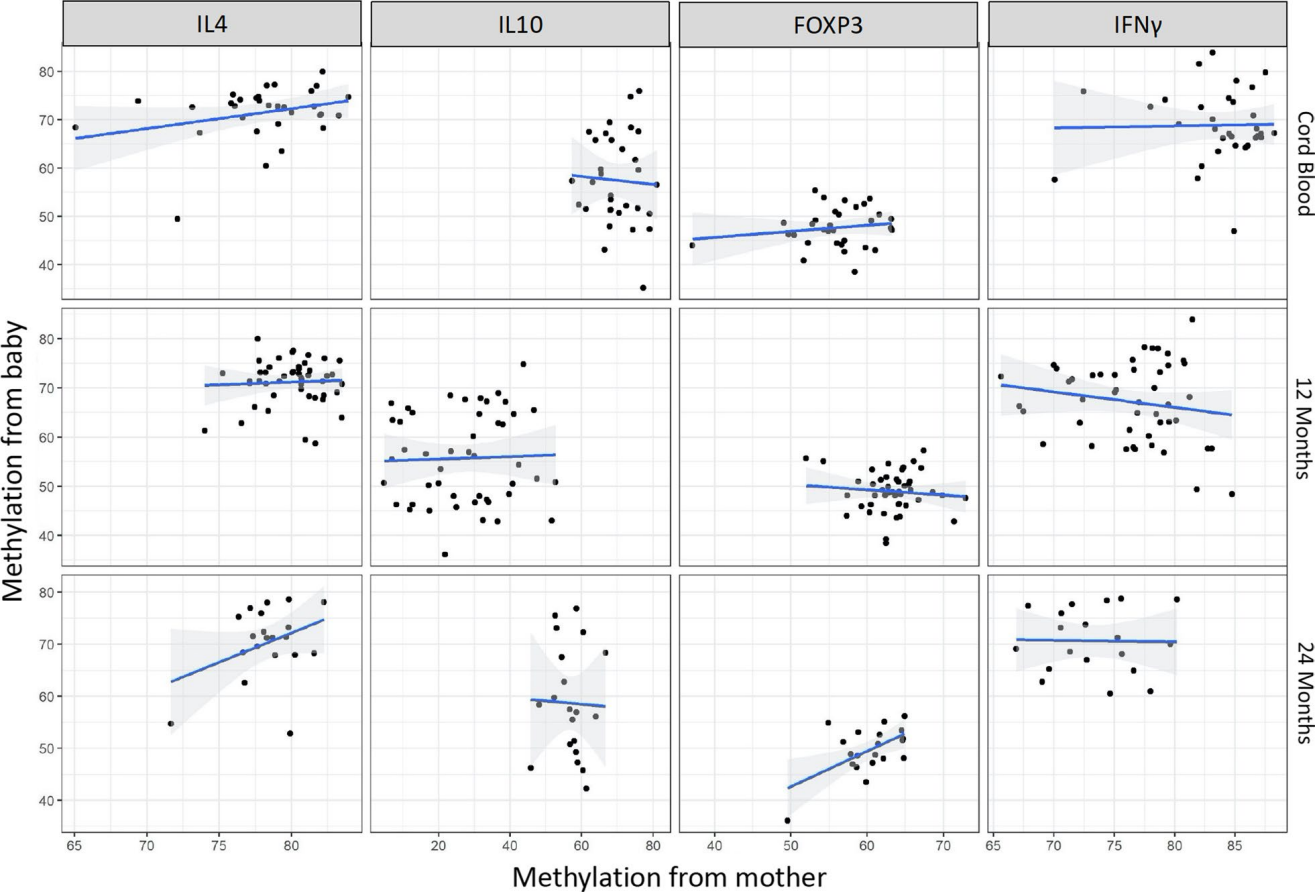
Associations between each pair of mother’s methylation and baby’s cord blood, at 12 months and 24 months. Bivariate analysis using Pearson’s correlation. False discovery rate adjustment at 0.1 significance level was applied

To determine whether the methylation level is driven by sub-cell type frequency we did further data analysis on the association between DNA methylation and sub-cell type frequency (Additional file 14: Fig. S14).There was an association between Th1 cell frequency and methylation level of the *IFNγ* gene regions, but the Th1 cell frequency was also associated with *IL4* methylation. There was no significant association between methylation level of *IL4* gene region and the Th2 cell frequency. Also, the mother and baby’s *IL4* and *IL10* methylation levels were not associated, after univariate analysis and FDR adjustment at 0.1 significance level (Fig. 7). Pearson’s correlation coefficients and corresponding 95% confidence intervals between mother’s and baby’s methylation levels were summarized in Additional file 19: Table S4.

## Discussion

In this cohort of pregnant women from Fresno, California, a metropolitan area known for elevated AAP levels, we found levels of AAPs to be associated with alterations in both pro and anti-inflammatory cytokines as well as a decrease in some Th cell population subsets. In general, these associations were sustained over time for short-term and mid-term AAP exposure.

Methylation of CpG sites in the *IL4* and *IL10* genes is related to anti-inflammatory responses, while methylation of the *IFNγ* gene would alter proinflammatory responses. IL4 aids in the polarization of antigen-stimulated naïve Th cells into Th2 effector cells and propagates Th2 responses, while IL10 downregulates Th1 cells [19, 29]. Our results indicate that, across multiple time exposure periods, higher levels of CO and PM_2.5_ are positively associated with methylation of the *IL4* gene, while higher levels of CO, NO_2_, and PAH_456_ are positively associated with methylation of the *IL10* gene. While the literature on methylation of *IL4* and *IL10* genes during pregnancy is scarce, prior studies have demonstrated an association between exposure to AAPs with IL4 in mice [24] and with IL10 in humans during late pregnancy [23]. Also, associations per pollutant and time average considered were not consistent between *IL4* and *IL10* gene methylation. For example, *IL10* methylation was significantly associated with PAH_456_ but not with *IL4* methylation. This is similar to another study where high levels of PAH_456_ were associated with decreases in protein expression of the *IL10* gene from 24 h to 1-year exposure, but not the *IL4* gene [30]. However, we also found that exposures to CO and PAH_456_ were associated with the methylation of *IFNγ,* which is pro-inflammatory. During the second trimester, these relationships could potentially be regulated by a gradual reversal of immune tolerance during mid to late pregnancy [10]. Furthermore, methylation of the *FoxP3* gene was only associated with exposure to PM_10_.

Exposures to PM_2.5,_ NO_2,_ and NO_X_ were inversely associated with Th2 cell percentages from 1 week to 6 months before the blood draw, while Th1 cells were inversely associated with CO, NO_x_, and PAH_456_ exposure from 1 week to 3 months. Th17 cells were also inversely associated with exposure to CO, NO_2_, NO_x_, PAH_456_, and PM_2.5_. Despite the relevance of these findings, there is scarce research on the direct effect of AAPs directly on T cell subsets during pregnancy. In murine models, PM and NO_2_ exposures exacerbate airway inflammation creating a Th1/Th2 imbalance [31]. Sasaki et al. reported that air pollutants can affect signaling pathways and expression of T cells associated with reduced expression of the *IFNγ* and *IL10* perturbing the Th1/Th2 balance [32]. Finally, PAH_456_ molecules within air pollution may play a role in the maintenance and function of Th17 responses [33].

Epigenetic marks may serve as biomarkers that can be used to support the prevention of adverse pregnancy events. The novelty of this study is the identification of multiple sites within a gene as well as gene regions sensitive to AAP exposures. Additional relationships with specific gene regions are available (Additional file 5: Fig. S5–Additional file 17: Fig. S17). Some of our findings were consistent with our a priori hypothesis, however, for O_3,_ we found associations contrary to our hypothesis. This may be due to the chemical equilibrium between ambient concentrations of O_3_ and NO_2_ such that local increases in NO_2_ are invariably accompanied by local decreases in O_3_ [34].

We did not find any association between the methylation of the mother’s blood and the baby’s cord blood or peripheral blood from 12 or 24 months. This is consistent with the findings by Friedman and colleagues who did not find consistent associations between infant outcomes and inflammatory biomarkers in the mother [35]. It is possible that individual genotypes at specific loci may result in different patterns of DNA methylation, which could influence extended genomic regions [36]. These methylation quantitative trait loci (meQTLs) may be responsible for direct gene-environment interactions which can vary over time under different conditions, such as exposure to AAPs.

To our knowledge, this is the first study to evaluate the association between T cell subsets and AAP exposure along with *IL4, IL10, IFNγ*, and *FoxP3* gene methylation in a sample of women at mid-pregnancy (20–23 weeks). Previous studies have not specified a critical time of exposure duration for effects on gene methylation or immune phenotype changes. However, our findings suggest some of the associations are sustained during pregnancy across the 6-month period before the blood draw.

One limitation is that the blood draws occurred across different seasons. To address this, we accounted for season, the distribution of subjects, and the spatial variation in pollutants in our statistical analysis plan. The 6-month exposure could potentially serve as a reference that could account for seasonal variations and could explain some of the unexpected trends for PM_10_. The estimated individual exposures to AAPs were assessed using modeling methods, rather than personal monitoring, so some exposure misclassification is likely. Also, to determine whether the methylation level could be driven by sub-cell type frequency, we performed further data analyses on the possible association between DNA methylation and sub-cell type frequency (Additional file 18: Fig. S18). There was no significant association between methylation levels of tested *IL4* gene regions and the Th2 cell frequency. There was an association between Th1 cell frequency and methylation level of the *IFNγ* gene region. Therefore, we cannot rule out the possibility that the sub-cell proportions may be a potential confounder.

Other limitations include lack of adjustment for dietary exposure and exposure to other toxicants from ambient/indoor air, water, and other sources which could contribute to DNA methylation and potentially confound results. The population considered was a convenience sample predominantly of Hispanic descent. Asthma status was self-reported by the participants rather than measured, but studies have shown good agreement between patient self-report and physician clinical diagnosis [37, 38].

In summary, we demonstrate that methylation levels at specific CpG sites in the *IL4*, *IL10*, and *IFNγ* genes are associated with exposure to the pollutants CO, NO_2_, PM_2.5_, and PAHs sustained up to 6 months before the blood draw. Also, the percent of Th1, Th2, and Th17 cells was negatively associated with several AAPs (CO, NO_2_, NOx, PAH_456_, and PM_2.5_). The findings from this study increase our understanding of the epigenetic effects of exposure to AAPs and may impact the development of therapies to alter the methylation of these sites. With the development of new therapies targeting specific gene sites and improved efforts to reduce exposure to ambient air pollutants, we aim to mitigate the impacts associated with AAP exposure during pregnancy, thereby reducing adverse health outcomes for both mother and baby.

## Conclusion

These results offer insights into the detrimental effects of air pollution during pregnancy, elucidating the role of epigenetic biomarkers and their possible uses for preventing or predicting epigenetic damage resulting from air pollution exposure.

## Methods

### Subject recruitment and study center visit

From November 2014 to August 2016, we recruited a convenience sample of 186 pregnant women from Fresno, California (median age = 27 years). Recruitment was done in cooperation with UCSF-Fresno-affiliated and Clinica Sierra Vista obstetric clinics, and the pregnant women were approached to participate in the clinic waiting areas by our field office staff.

Interested women were screened for eligibility (between 18 and 25 weeks gestation, residence in Fresno or Clovis for at least the past 3 months, residence within 20 km of the central air quality monitoring site, no plans to move from the Fresno/Clovis area in the next 2 years, English- or Spanish-speaking, smoked < 50 cigarettes during their pregnancy, and no cancer, HIV, or autoimmune disease). If interested and eligible, the pregnant women were invited to visit the study center at UCSF-Fresno. All study protocols were approved by the Institutional Review Boards at the University of California, Berkeley; the University of California, San Francisco-Fresno (UCSF-Fresno); and Stanford University. Written, informed permission was obtained from each subject.

During each visit, we collected information on height and weight, a health and general history questionnaire, and blood samples. We ensured that subjects that took oral immunosuppressants within 5 days of the blood draw, had a history of allergen immunotherapy within 1 year of the visit, had a chronic disease other than allergies or asthma, or had an acute infection were not considered in the analysis.

Cord blood was collected at birth from a convenience sample (n = 35) of enrolled mothers who were invited to enroll their babies and come in for office visits when the babies were approximately 12- (n = 58) and 24-months old (n = 20). After the mothers gave written permission for their babies to participate in the study, we collected information for the babies on length and weight, conducted a health and general history questionnaire, and also took blood samples from a convenience sample of the babies to explore methylation associations between mother-baby pairs at each of three time points: birth (cord blood), 12 months and 24 months. We did not include samples from twin births.

### Collection and processing of blood specimens

We extracted human peripheral blood mononuclear cells (PBMCs) and plasma from the collected blood sample using a Ficoll procedure which allows rapid and efficient isolation of mononuclear cells and stored them in liquid nitrogen and at − 80 °C as detailed in previous research [39].

### Methylation sequencing and analysis

We extracted DNA from frozen PBMCs using the QIAamp DNA blood Mini Kit (Qiagen, Valencia, CA) which was subjected to a bisulfite modification through the EZ DNA Methylation Kit (Zymo Research, Orange, CA) based on manufacturer recommendations as described in published works [25]. We conducted a methylation analysis on *IL4, IL10, FoxP3*, and *IFNγ* genes. Based on previous findings, we selected key sites of important immunoregulatory genes that modulate immune tolerance [40–42]. We selected 6 CpG sites in the intron 2 region of the *IL4* gene (Chr5:132673938, Chr5:132673907, Chr5:132675095, Chr5:132675115, Chr5:132675133, Chr5:132675242) and 4 CpG sites in the intron 4 region of the *IL10* gene (Chr1:206769266, Chr1:206769234, Chr1:206769230, Chr1:206769214), respectively. We also selected 2 CpG sites in the promoter region of the *FoxP3* gene (ChrX:49264916, ChrX:49264956) and 3 CpG sites in the promoter region of the *IFNγ* gene (Chr12:68159798, Chr12:68159930, Chr12:68160040). We included internal controls for bisulfite conversion efficiency in each pyrosequencing assay and followed standardized operating procedures (SOPs) and quality control from the Sean Parker Center for Allergy and Asthma Research’s laboratory as done in previous work [43]. We explored associations between AAP exposure (CO, O_3_, NO_2_, NOx, PAH_456_, PM_2.5,_ and PM_10_) and methylation levels at the aforementioned CpG sites.

### Flow cytometry

We also considered the frequency of different T helper (Th1, Th2, Th17, and Treg) subset populations using flow cytometry. We resuspended the cells in PBS in flow-cytometry staining tubes. We used live/dead staining (Molecular Probes/Invitrogen) in all our samples and identified live cells by the intracellular conversion of a calcein ester to free calcein, which is fluorescent in the green spectrum; we identified dead cells by the red staining of internal nucleic acids using ethidium homodimer. We further stained the fraction with CD4-Pacific Blue (clone RPA-T4, BD Biosciences), CD25-phycoerythrin (PE)-Cy7 (clone BC96, BioLegend), and CD127-FITC (BD Biosciences). We gated CD127 low-expressing CD4 + CD25hi cells to ensure separation of conventional activated T cells from stable Treg populations, which further allowed us to identify highly-enriched FoxP3 + cells [25].

### Ambient air pollution exposure assessment

Average air pollutant exposures were calculated for each mother over several different time periods: 1 week, and 1, 3 and 6 months before the blood sample was collected. To calculate air pollution exposures, we modeled outdoor residential air pollution exposure to AAPs, specifically CO, O_3_, NO_2_, NOx, EC, PAH_456_, PM_2.5,_ and PM_10_ (PAH_456_ is defined as polycyclic aromatic hydrocarbons with 4, 5 and 6 rings (fluoranthene, benz[a]anthracene, chrysene, benzo[a]pyrene, benzo[b]fluoranthene, benzo[k]flouoranthene, benzo[ghi]perylene, indeno[1,2,3-cd]pyrene, and dibenz[a,h]anthracene). Two methods were used for individual-level exposure assessment depending on the specific pollutant of interest: interpolation of data extracted from the US Environmental Protection Agency’s (EPA) Air Quality System using inverse distance-squared weighting for CO, NO_2_, NOx, O_3_, PM_2.5,_ and PM_10_, and regression modeling incorporating monitoring data and field sampling data for EC, and PAH_456_.

EPA’s Air Quality System offered hourly, quality-assured, ambient pollutant concentrations and meteorological data from Fresno’s site monitoring stations. Elemental carbon (BC) was determined from Aethalometer™ (model AE42; Magee Scientific, Berkeley, CA) measurements of the optical absorption of PM_2.5_ ambient aerosol at 880 nm. Particle-bound PAHs were monitored with the PAS 2000 (EcoChem Analytics, League City, TX) which uses a photoelectric aerosol sensor to measure ambient PAH with three or more rings. Data from these real-time continuous monitors are available from 2002 through 2017 covering the time window of our study. The air pollution data were subject to rigorous quality assurance which included the comparison of values at nearby sites and consistency with historical patterns for each pollutant. These methods have been described in detail elsewhere [44–46]. Lastly, a residential address history was obtained from each participating mother, and exposure was matched to participants’ residential street addresses.

### Statistical analysis

The DNA methylation was calculated as the average of the selected CpG sites for each gene. To evaluate the associations between gene methylation and air pollution, we constructed a linear regression model on each methylation as a function of each AAP exposure, adjusting for weight, age, season, race, and asthma. The *p*-values for the association with exposure across all genes were adjusted for multiple testing using the Benjamini–Hochberg procedures that control the false-discovery rate (FDR). The selection of covariates is based on our previous epigenetic work [26, 27]. Seasons were defined as described in previous work [47]. For race, the respondents were allowed to select up to 4 race/ethnicity responses for themselves, which were upcoded to Hispanic, Black/African American, Non-Hispanic White, Asian/Pacific Islander, and American Indian/Alaska Native. Any subject coded as Hispanic in any reported category was defined as Hispanic, and among those remaining, the first other listed race was used. We further constructed the same model for each CpG site as a supplementary analysis. We adjusted the *p-*values of exposures across all CpG sites for multiple testing using the same method. The correlations between mother’s and cord blood, and with baby’s methylation levels at 12 months and 24 months were assessed using Pearson’s correlation coefficients; *p*-values across all genes were adjusted for multiple testing using the same method. All tests were two-sided. We used a *Q* value to denote the adjusted *p*-value, and *Q* < 0.1 was considered statistically significant. The statistical analysis was performed using the R statistical computing software (R 3.4.1).

## Supplementary Information

**Additional file 1: Figure S1**. Ambient air pollutant (AAP) concentration levels and counts per participant. CO: Carbon monoxide, EC: Elemental carbon, NO_2_: Nitric dioxide, NOx: Nitric oxides, O_3_: Ozone, PAH: Polycyclic aromatic hydrocarbons, PM: particulate matter.

**Additional file 2: Figure S2**. Associations between Foxp3 and Ambient Air Pollutant levels. *Q* value is the false-discovery-rate-adjusted p-value across all genes, based on linear regression model adjusting for weight, age, season, race, and asthma diagnosis. *Q* < 0.1 is considered statistically significant. FoxP3 refers to average DNA methylation across 3 CpG sites in the gene. CO: Carbon monoxide, EC: Elemental carbon, NO_2_: Nitric dioxide, NOx: Nitric oxides, O_3_: Ozone, PAH: Polycyclic aromatic hydrocarbons, PM: particulate matter.

**Additional file 3: Figure S3**. Associations between Treg percentage and Ambient Air Pollutant levels. *Q* value is the false-discovery-rate-adjusted p-value across all genes, based on linear regression model adjusting for weight, age, season, race, and asthma diagnosis. *Q* < 0.1 is considered statistically significant. CO: Carbon monoxide, EC: Elemental carbon, NO_2_: Nitric dioxide, NOx: Nitric oxides, O_3_: Ozone, PAH: Polycyclic aromatic hydrocarbons, PM: particulate matter.

**Additional file 4: Figure S4**. Associations between each T cell sublet percentage and each CpG site methylation. *Q* value is the false-discovery-rate-adjusted p-value across all genes, based on linear regression model adjusting for weight, age, season, race, and asthma diagnosis. *Q* < 0.1 is considered statistically significant.

**Additional file 5: Figure S5**. Associations between IL4_CpG4 site and Ambient Air Pollutant levels. *Q* value is the false-discovery-rate-adjusted p-value across all genes, based on linear regression model adjusting for weight, age, season, race, and asthma diagnosis. *Q* < 0.1 is considered statistically significant. CO: Carbon monoxide, EC: Elemental carbon, NO_2_: Nitric dioxide, NOx: Nitric oxides, O_3_: Ozone, PAH: Polycyclic aromatic hydrocarbons, PM: particulate matter.

**Additional file 6: Figure S6**. Associations between IL4_CpG3 site and Ambient Air Pollutant levels. *Q* value is the false-discovery-rate-adjusted p-value across all genes, based on linear regression model adjusting for weight, age, season, race, and asthma diagnosis. *Q* < 0.1 is considered statistically significant. CO: Carbon monoxide, EC: Elemental carbon, NO_2_: Nitric dioxide, NOx: Nitric oxides, O_3_: Ozone, PAH: Polycyclic aromatic hydrocarbons, PM: particulate matter.

**Additional file 7: Figure S7**. Associations between IL4_CpG24 site and Ambient Air Pollutant levels. *Q* value is the false-discovery-rate-adjusted p-value across all genes, based on linear regression model adjusting for weight, age, season, race, and asthma diagnosis. *Q* < 0.1 is considered statistically significant. CO: Carbon monoxide, EC: Elemental carbon, NO_2_: Nitric dioxide, NOx: Nitric oxides, O_3_: Ozone, PAH: Polycyclic aromatic hydrocarbons, PM: particulate matter.

**Additional file 8: Figure S8**. Associations between IL4_CpG22 site and Ambient Air Pollutant levels. *Q* value is the false-discovery-rate-adjusted p-value across all genes, based on linear regression model adjusting for weight, age, season, race, and asthma diagnosis. *Q* < 0.1 is considered statistically significant. CO: Carbon monoxide, EC: Elemental carbon, NO_2_: Nitric dioxide, NOx: Nitric oxides, O_3_: Ozone, PAH: Polycyclic aromatic hydrocarbons, PM: particulate matter.

**Additional file 9: Figure S9**. Associations between IL4_CpG21 site and Ambient Air Pollutant levels. *Q* value is the false-discovery-rate-adjusted p-value across all genes, based on linear regression model adjusting for weight, age, season, race, and asthma diagnosis. *Q* < 0.1 is considered statistically significant. CO: Carbon monoxide, EC: Elemental carbon, NO_2_: Nitric dioxide, NOx: Nitric oxides, O_3_: Ozone, PAH: Polycyclic aromatic hydrocarbons, PM: particulate matter.

**Additional file 10: Figure S10**. Associations between IL4_lossCpG23 site and Ambient Air Pollutant levels. *Q* value is the false-discovery-rate-adjusted p-value across all genes, based on linear regression model adjusting for weight, age, season, race, and asthma diagnosis. *Q* < 0.1 is considered statistically significant. CO: Carbon monoxide, EC: Elemental carbon, NO_2_: Nitric dioxide, NOx: Nitric oxides, O_3_: Ozone, PAH: Polycyclic aromatic hydrocarbons, PM: particulate matter.

**Additional file 11: Figure S11**. Associations between IL10_CpG38 site and Ambient Air Pollutant levels. *Q* value is the false-discovery-rate-adjusted p-value across all genes, based on linear regression model adjusting for weight, age, season, race, and asthma diagnosis. *Q* < 0.1 is considered statistically significant. CO: Carbon monoxide, EC: Elemental carbon, NO_2_: Nitric dioxide, NOx: Nitric oxides, O_3_: Ozone, PAH: Polycyclic aromatic hydrocarbons, PM: particulate matter.

**Additional file 12: Figure S12**. Associations between IL10_CpG39 site and Ambient Air Pollutant levels. *Q* value is the false-discovery-rate-adjusted p-value across all cell types, based on linear regression model adjusting for weight, age, season, race, and asthma diagnosis. *Q* < 0.1 are shown. CO: Carbon monoxide, EC: Elemental carbon, NO_2_: Nitric dioxide, NOx: Nitric oxides, O_3_: Ozone, PAH: Polycyclic aromatic hydrocarbons, PM: particulate matter.

**Additional file 13: Figure S13**. Associations between IL10_CpG40 site and Ambient Air Pollutant levels. *Q* value is the false-discovery-rate-adjusted p-value across all genes, based on linear regression model adjusting for weight, age, season, race, and asthma diagnosis. *Q* < 0.1 is considered statistically significant. CO: Carbon monoxide, EC: Elemental carbon, NO_2_: Nitric dioxide, NOx: Nitric oxides, O_3_: Ozone, PAH: Polycyclic aromatic hydrocarbons, PM: particulate matter.

**Additional file 14: Figure S14**. Associations between IL10_CpG41 site and Ambient Air Pollutant levels. *Q* value is the false-discovery-rate-adjusted p-value across all genes, based on linear regression model adjusting for weight, age, season, race, and asthma diagnosis. *Q* < 0.1 is considered statistically significant. CO: Carbon monoxide, EC: Elemental carbon, NO_2_: Nitric dioxide, NOx: Nitric oxides, O_3_: Ozone, PAH: Polycyclic aromatic hydrocarbons, PM: particulate matter.

**Additional file 15: Figure S15**. Associations between IFNγ_CpG5 site and Ambient Air Pollutant levels. *Q* value is the false-discovery-rate-adjusted p-value across all cell types, based on linear regression model adjusting for weight, age, season, race, and asthma diagnosis. *Q* < 0.1 are shown. CO: Carbon monoxide, EC: Elemental carbon, NO_2_: Nitric dioxide, NOx: Nitric oxides, O_3_: Ozone, PAH: Polycyclic aromatic hydrocarbons, PM: particulate matter.

**Additional file 16: Figure S16**. Associations between IFNγ_CpG4 site and Ambient Air Pollutant levels. *Q* value is the false-discovery-rate-adjusted p-value across all genes, based on linear regression model adjusting for weight, age, season, race, and asthma diagnosis. *Q* < 0.1 is considered statistically significant. CO: Carbon monoxide, EC: Elemental carbon, NO_2_: Nitric dioxide, NOx: Nitric oxides, O_3_: Ozone, PAH: Polycyclic aromatic hydrocarbons, PM: particulate matter.

**Additional file 17: Figure S17**. Associations between IFNγ_CpG3 site and Ambient Air Pollutant levels. *Q* value is the false-discovery-rate-adjusted p-value across all genes, based on linear regression model adjusting for weight, age, season, race, and asthma diagnosis. *Q* < 0.1 is considered statistically significant. CO: Carbon monoxide, EC: Elemental carbon, NO_2_: Nitric dioxide, NOx: Nitric oxides, O_3_: Ozone, PAH: Polycyclic aromatic hydrocarbons, PM: particulate matter.

**Additional file 18: Figure S18**. Associations between each cell type percentage vs each gene. *Q* value is the false-discovery-rate-adjusted p-value across all genes, based on linear regression model adjusting for weight, age, season, race, and asthma diagnosis. *Q* < 0.1 is considered statistically significant.

**Additional file 19:** Supplementary file with tables of (1) flow cytometry panel analysis, (2) summary of CpG site of the 4 genes Foxp3, Il-4, Il-10 and IFN-γ, (3) estimates and coefficients of gene and pollutant data by time estimate, and (4) estimates and correlation coefficients of mother and baby data.

## Data Availability

The datasets used and/or analyzed during the current study are available from the corresponding author on reasonable request.
