# Supplementary figures and images for: Increases in ambient air pollutants during pregnancy are linked to increases in methylation of IL4, IL10, and IFNγ
Source: Clin Epigenetics. 2022 Mar 14;14:40. doi: 10.1186/s13148-022-01254-2 (PMC8919561; doi:10.1186/s13148-022-01254-2)

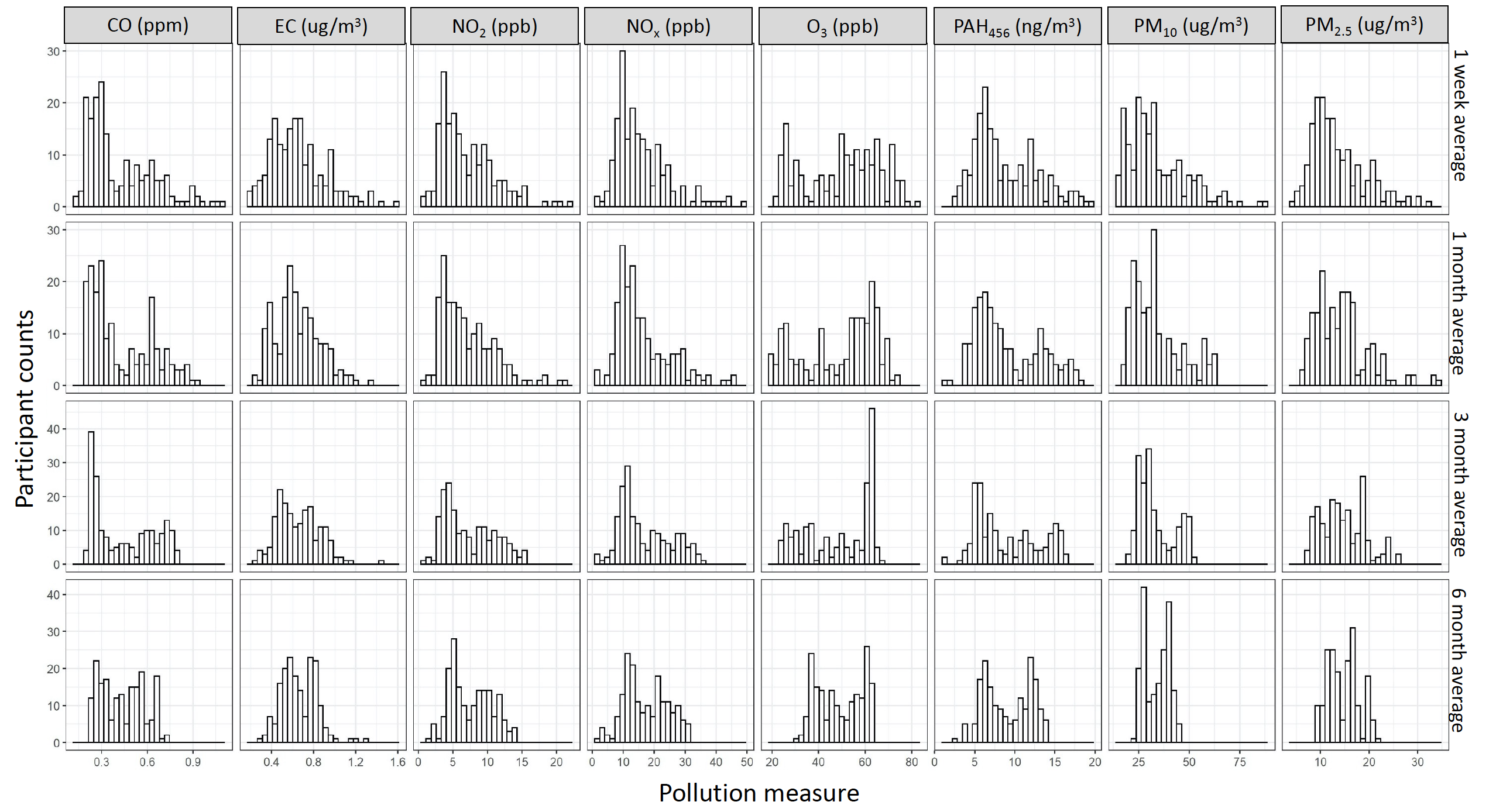

Supplement: Supplementary file 1 — Additional file 1: Figure S1. Ambient air pollutant (AAP) concentration levels and counts per participant. CO: Carbon monoxide, EC: Elemental carbon, NO2: Nitric dioxide, NOx: Nitric oxides, O3: Ozone, PAH: Polycyclic aromatic hydrocarbons, PM: particulate matter. [file 13148_2022_1254_MOESM1_ESM.png]

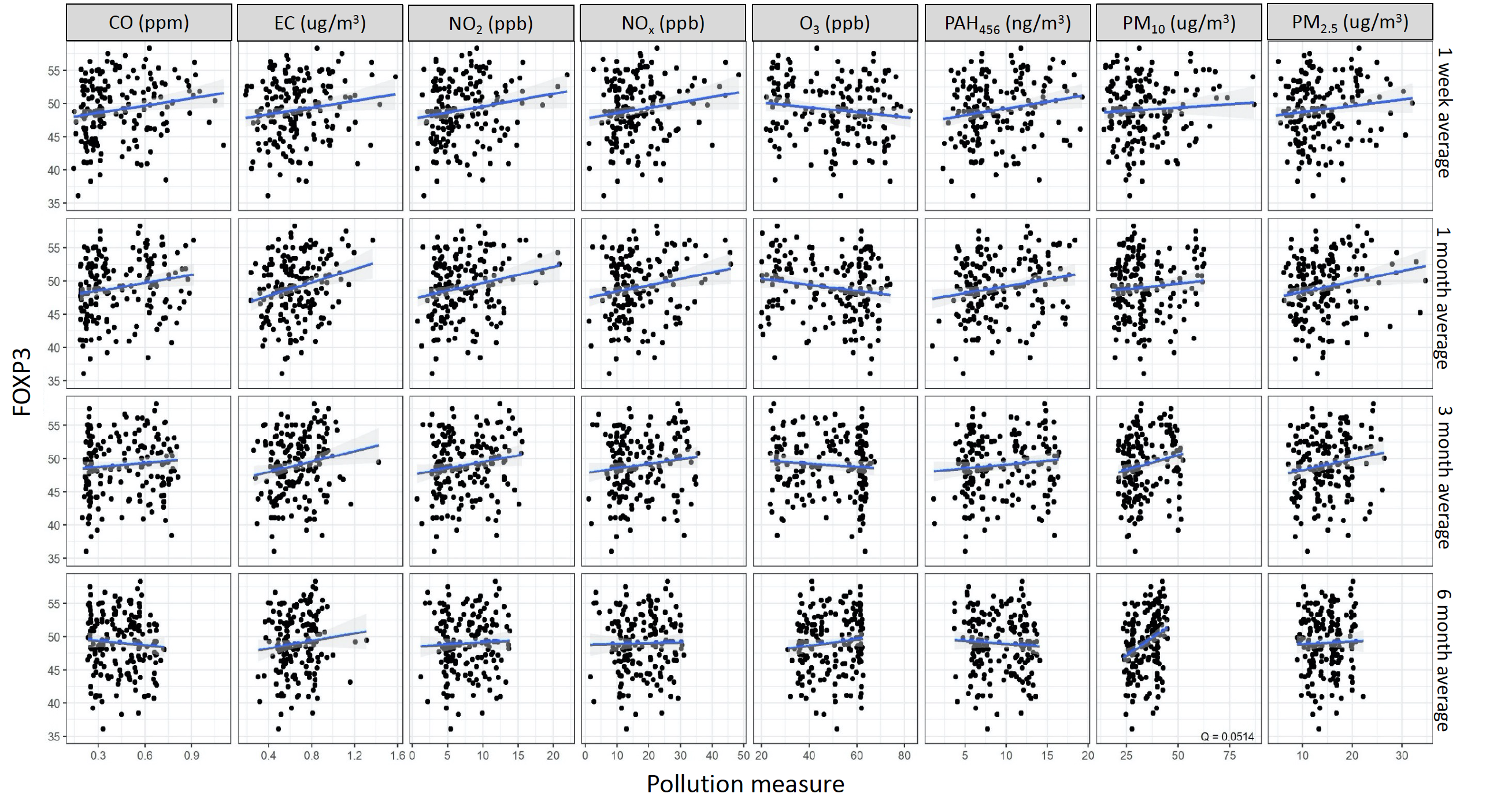

Supplement: Supplementary file 2 — Additional file 2: Figure S2. Associations between Foxp3 and Ambient Air Pollutant levels. Q value is the false-discovery-rate-adjusted p-value across all genes, based on linear regression model adjusting for weight, age, season, race, and asthma diagnosis. Q < 0.1 is considered statistically significant. FoxP3 refers to average DNA methylation across 3 CpG sites in the gene. CO: Carbon monoxide, EC: Elemental carbon, NO2: Nitric dioxide, NOx: Nitric oxides, O3: Ozone, PAH: Polycyclic aromatic hydrocarbons, PM: particulate matter. [file 13148_2022_1254_MOESM2_ESM.png]

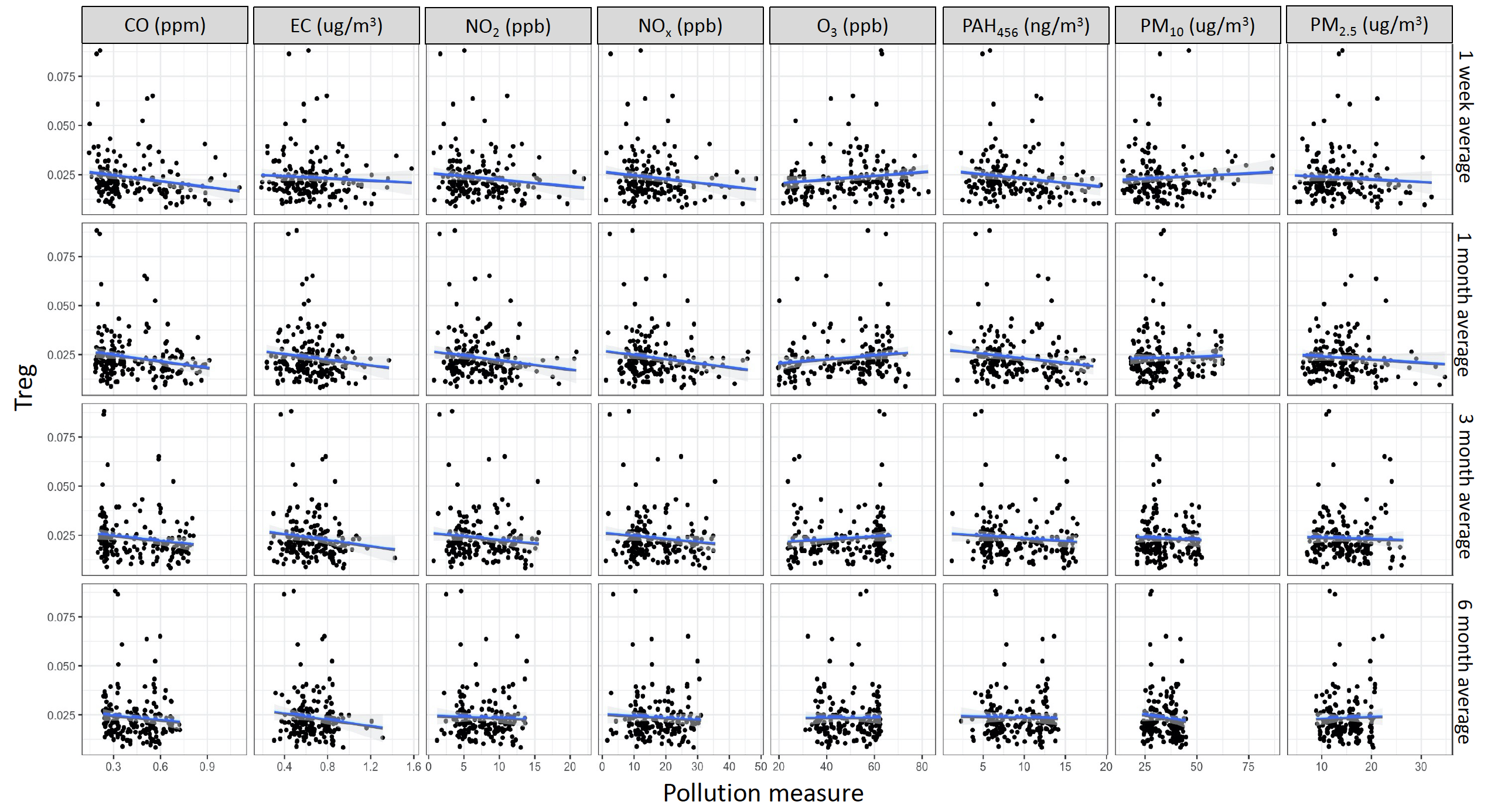

Supplement: Supplementary file 3 — Additional file 3: Figure S3. Associations between Treg percentage and Ambient Air Pollutant levels. Q value is the false-discovery-rate-adjusted p-value across all genes, based on linear regression model adjusting for weight, age, season, race, and asthma diagnosis. Q < 0.1 is considered statistically significant. CO: Carbon monoxide, EC: Elemental carbon, NO2: Nitric dioxide, NOx: Nitric oxides, O3: Ozone, PAH: Polycyclic aromatic hydrocarbons, PM: particulate matter. [file 13148_2022_1254_MOESM3_ESM.png]

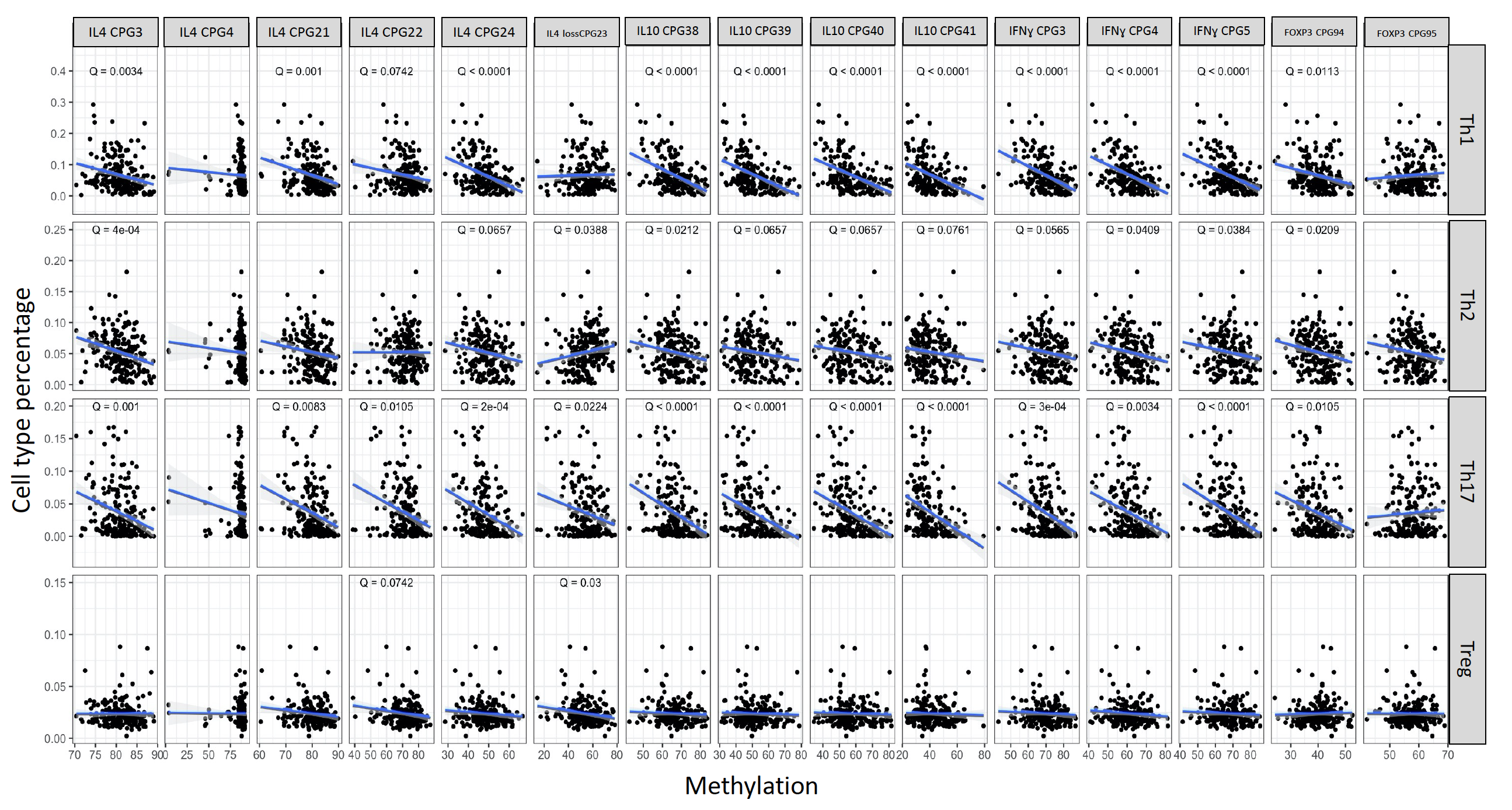

Supplement: Supplementary file 4 — Additional file 4: Figure S4. Associations between each T cell sublet percentage and each CpG site methylation. Q value is the false-discovery-rate-adjusted p-value across all genes, based on linear regression model adjusting for weight, age, season, race, and asthma diagnosis. Q < 0.1 is considered statistically significant. [file 13148_2022_1254_MOESM4_ESM.png]

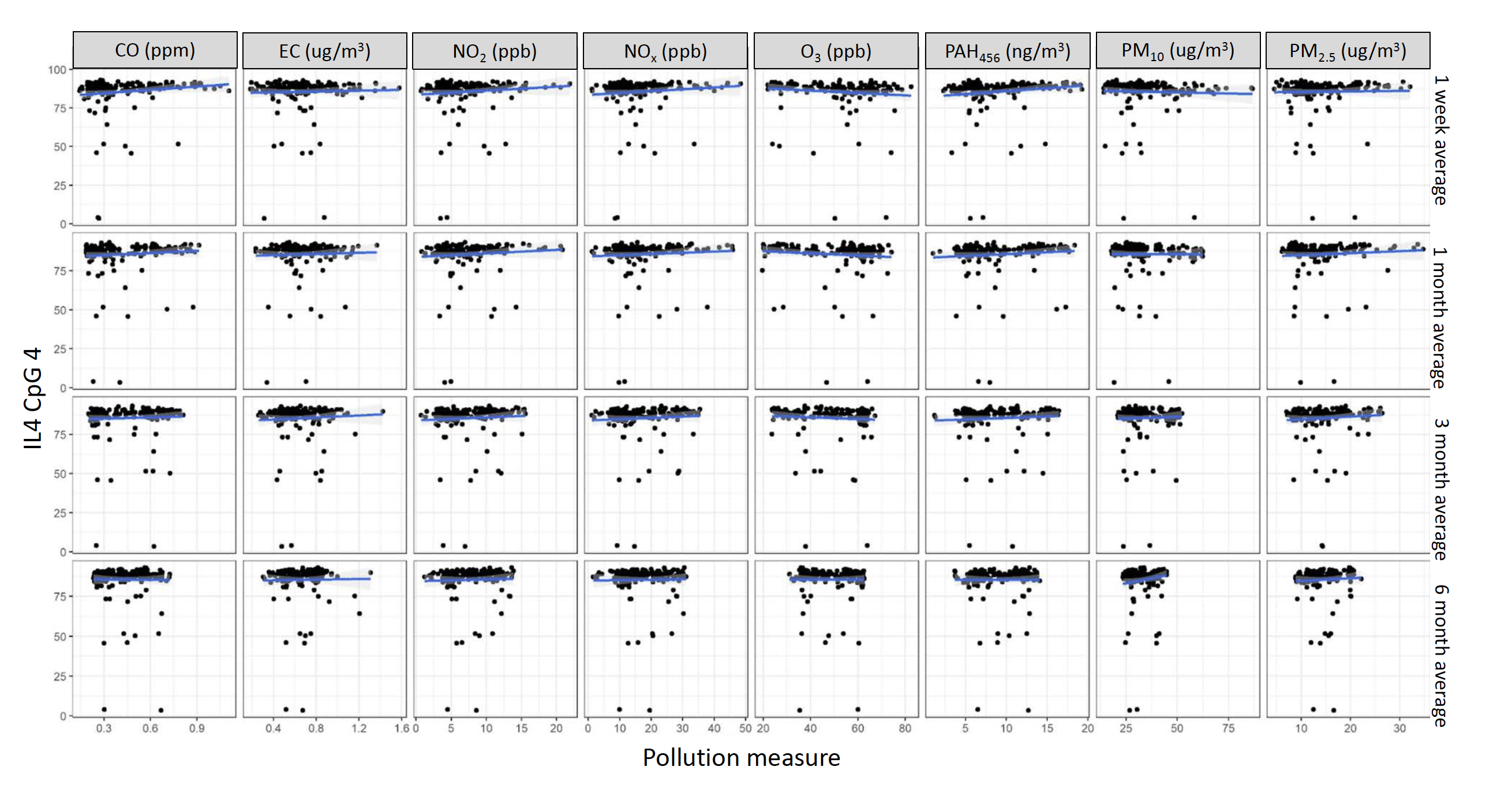

Supplement: Supplementary file 5 — Additional file 5: Figure S5. Associations between IL4_CpG4 site and Ambient Air Pollutant levels. Q value is the false-discovery-rate-adjusted p-value across all genes, based on linear regression model adjusting for weight, age, season, race, and asthma diagnosis. Q < 0.1 is considered statistically significant. CO: Carbon monoxide, EC: Elemental carbon, NO2: Nitric dioxide, NOx: Nitric oxides, O3: Ozone, PAH: Polycyclic aromatic hydrocarbons, PM: particulate matter. [file 13148_2022_1254_MOESM5_ESM.png]

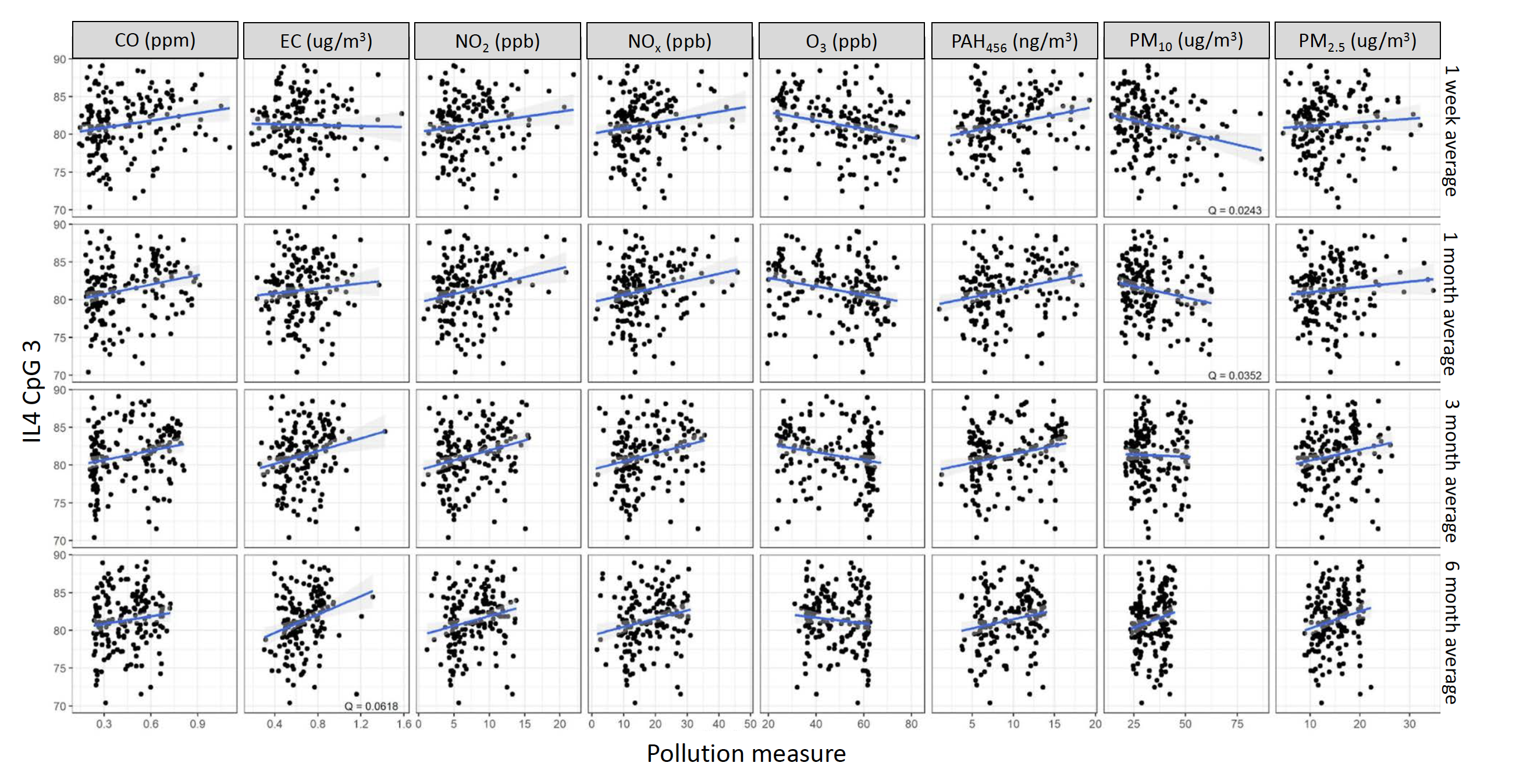

Supplement: Supplementary file 6 — Additional file 6: Figure S6. Associations between IL4_CpG3 site and Ambient Air Pollutant levels. Q value is the false-discovery-rate-adjusted p-value across all genes, based on linear regression model adjusting for weight, age, season, race, and asthma diagnosis. Q < 0.1 is considered statistically significant. CO: Carbon monoxide, EC: Elemental carbon, NO2: Nitric dioxide, NOx: Nitric oxides, O3: Ozone, PAH: Polycyclic aromatic hydrocarbons, PM: particulate matter. [file 13148_2022_1254_MOESM6_ESM.png]

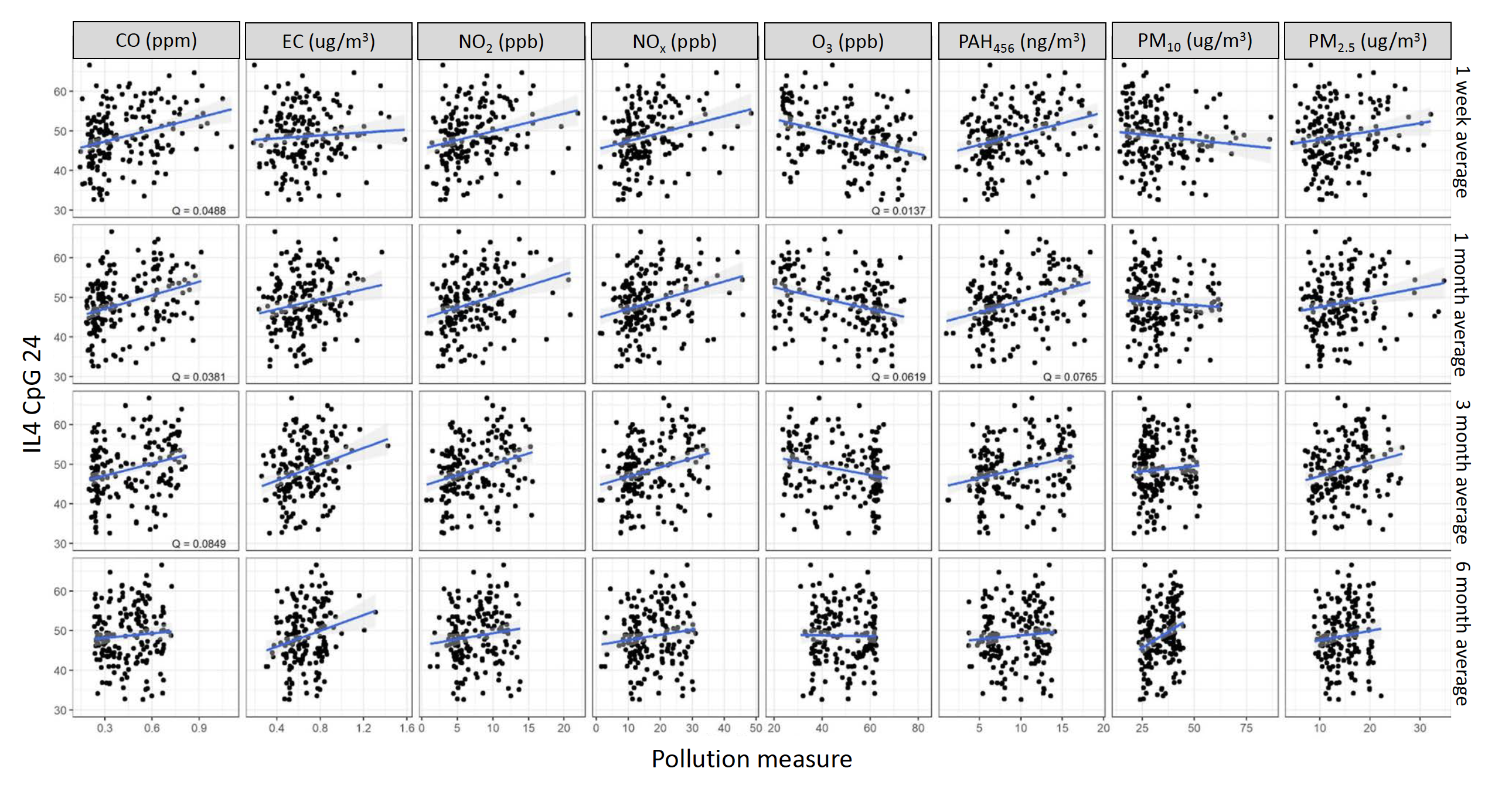

Supplement: Supplementary file 7 — Additional file 7: Figure S7. Associations between IL4_CpG24 site and Ambient Air Pollutant levels. Q value is the false-discovery-rate-adjusted p-value across all genes, based on linear regression model adjusting for weight, age, season, race, and asthma diagnosis. Q < 0.1 is considered statistically significant. CO: Carbon monoxide, EC: Elemental carbon, NO2: Nitric dioxide, NOx: Nitric oxides, O3: Ozone, PAH: Polycyclic aromatic hydrocarbons, PM: particulate matter. [file 13148_2022_1254_MOESM7_ESM.png]

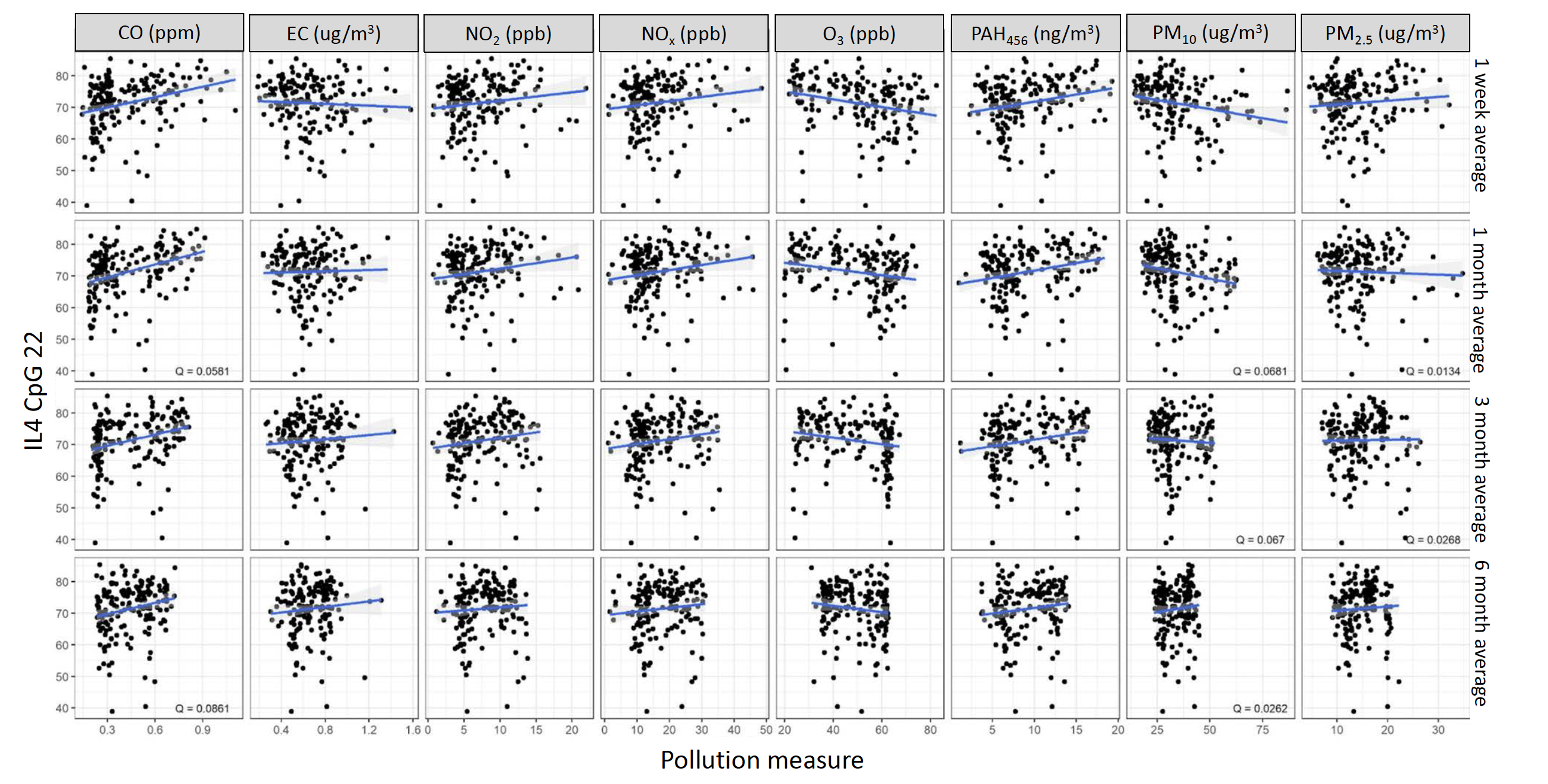

Supplement: Supplementary file 8 — Additional file 8: Figure S8. Associations between IL4_CpG22 site and Ambient Air Pollutant levels. Q value is the false-discovery-rate-adjusted p-value across all genes, based on linear regression model adjusting for weight, age, season, race, and asthma diagnosis. Q < 0.1 is considered statistically significant. CO: Carbon monoxide, EC: Elemental carbon, NO2: Nitric dioxide, NOx: Nitric oxides, O3: Ozone, PAH: Polycyclic aromatic hydrocarbons, PM: particulate matter. [file 13148_2022_1254_MOESM8_ESM.png]

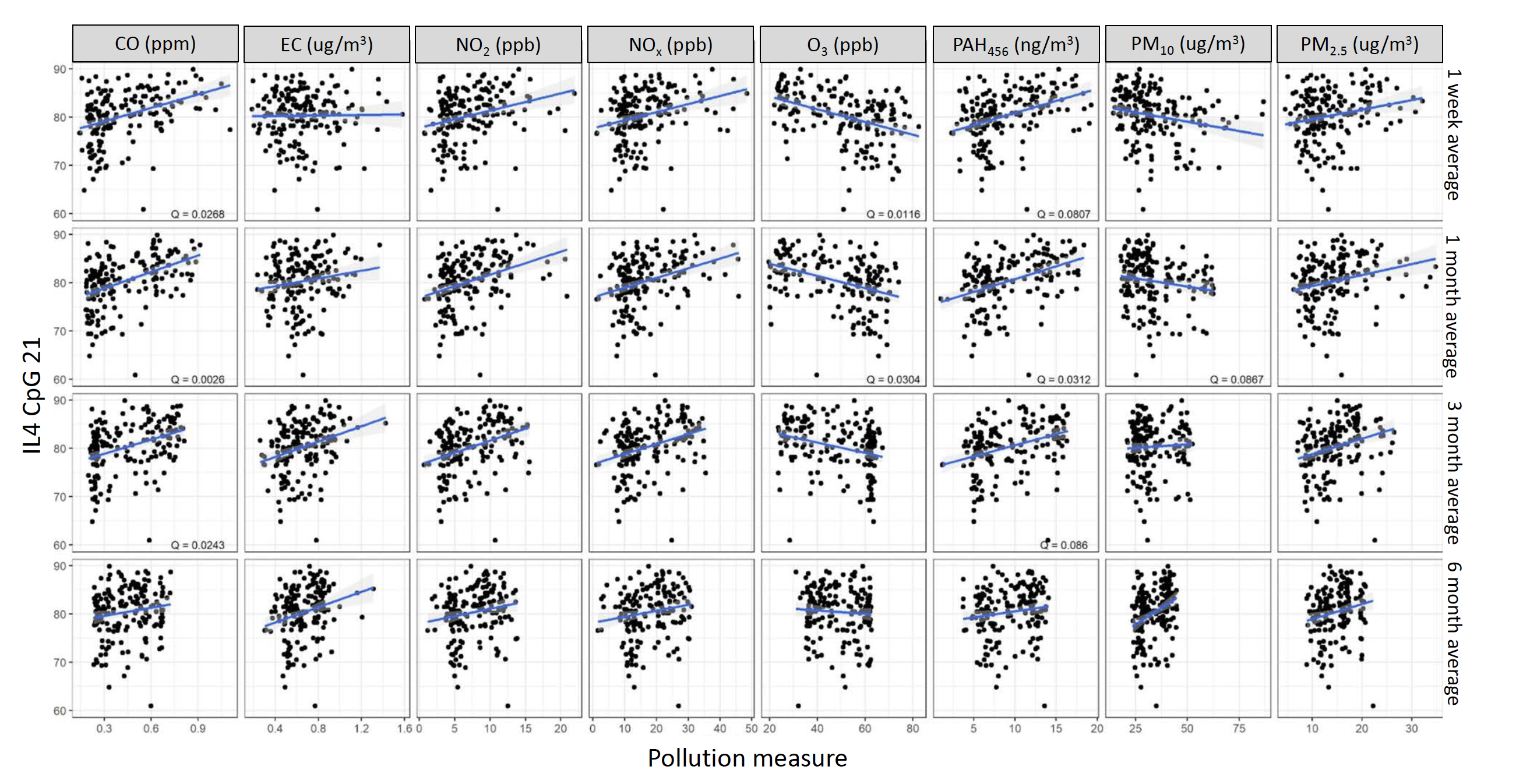

Supplement: Supplementary file 9 — Additional file 9: Figure S9. Associations between IL4_CpG21 site and Ambient Air Pollutant levels. Q value is the false-discovery-rate-adjusted p-value across all genes, based on linear regression model adjusting for weight, age, season, race, and asthma diagnosis. Q < 0.1 is considered statistically significant. CO: Carbon monoxide, EC: Elemental carbon, NO2: Nitric dioxide, NOx: Nitric oxides, O3: Ozone, PAH: Polycyclic aromatic hydrocarbons, PM: particulate matter. [file 13148_2022_1254_MOESM9_ESM.png]

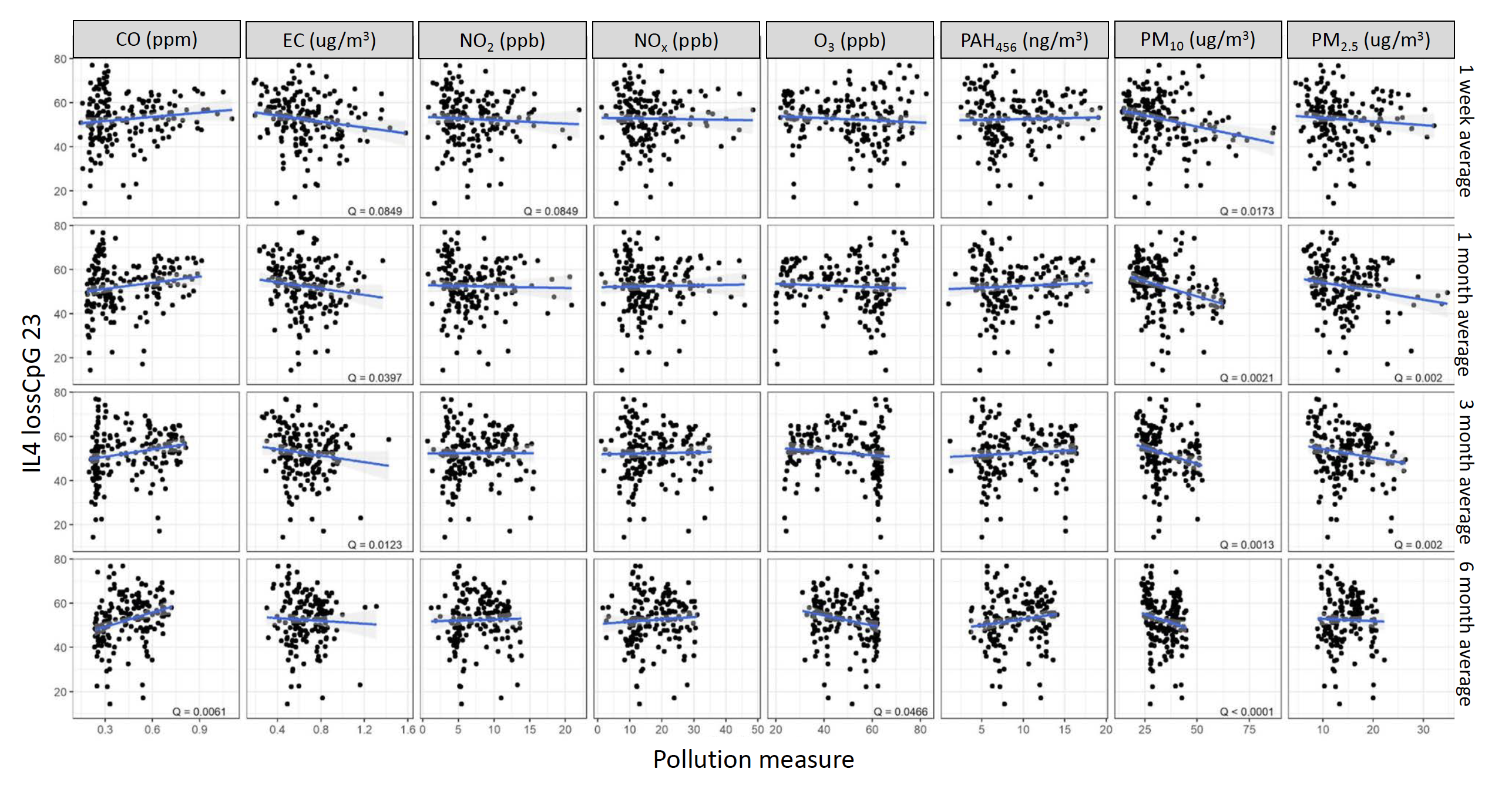

Supplement: Supplementary file 10 — Additional file 10: Figure S10. Associations between IL4_lossCpG23 site and Ambient Air Pollutant levels. Q value is the false-discovery-rate-adjusted p-value across all genes, based on linear regression model adjusting for weight, age, season, race, and asthma diagnosis. Q < 0.1 is considered statistically significant. CO: Carbon monoxide, EC: Elemental carbon, NO2: Nitric dioxide, NOx: Nitric oxides, O3: Ozone, PAH: Polycyclic aromatic hydrocarbons, PM: particulate matter. [file 13148_2022_1254_MOESM10_ESM.png]

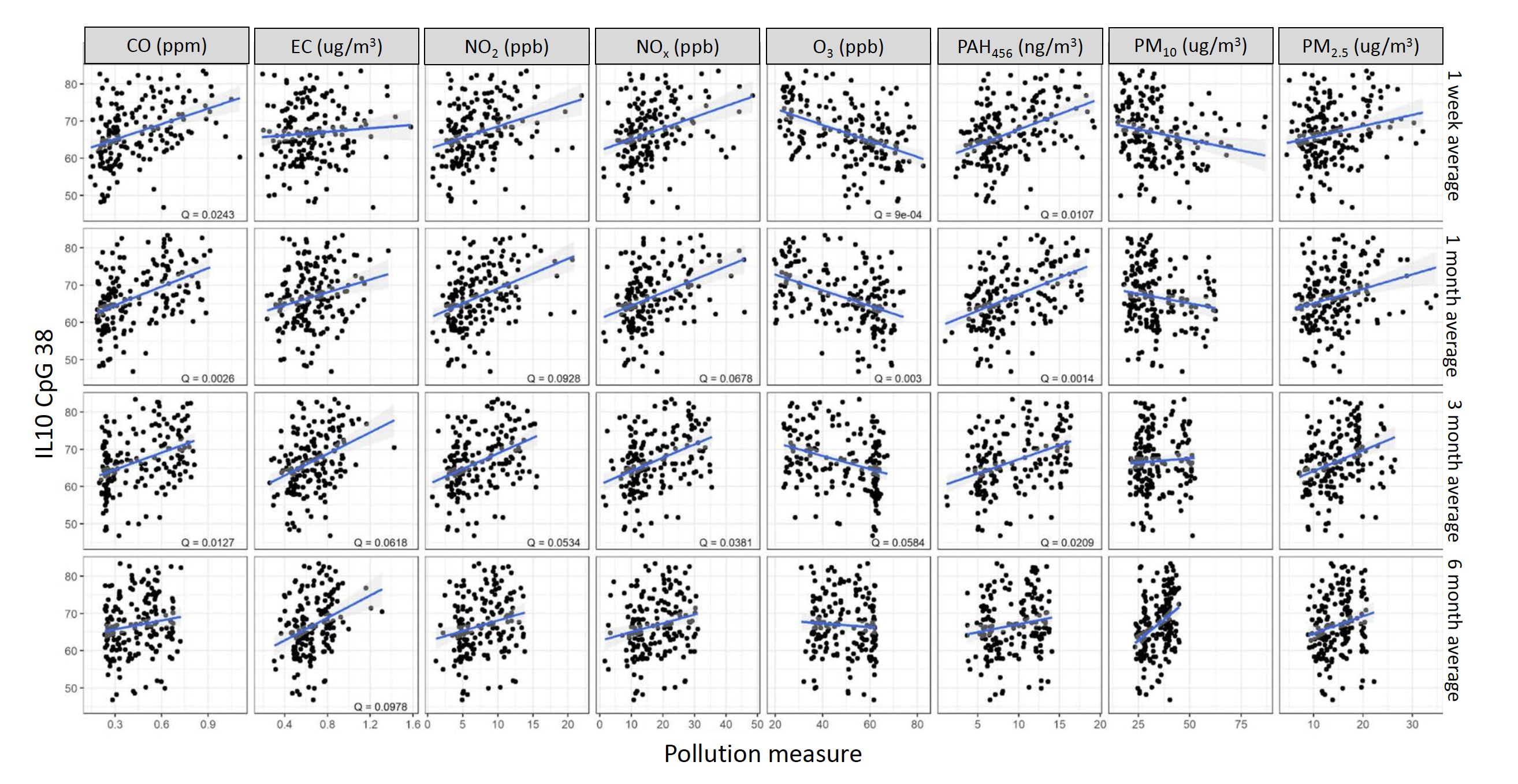

Supplement: Supplementary file 11 — Additional file 11: Figure S11. Associations between IL10_CpG38 site and Ambient Air Pollutant levels. Q value is the false-discovery-rate-adjusted p-value across all genes, based on linear regression model adjusting for weight, age, season, race, and asthma diagnosis. Q < 0.1 is considered statistically significant. CO: Carbon monoxide, EC: Elemental carbon, NO2: Nitric dioxide, NOx: Nitric oxides, O3: Ozone, PAH: Polycyclic aromatic hydrocarbons, PM: particulate matter. [file 13148_2022_1254_MOESM11_ESM.png]

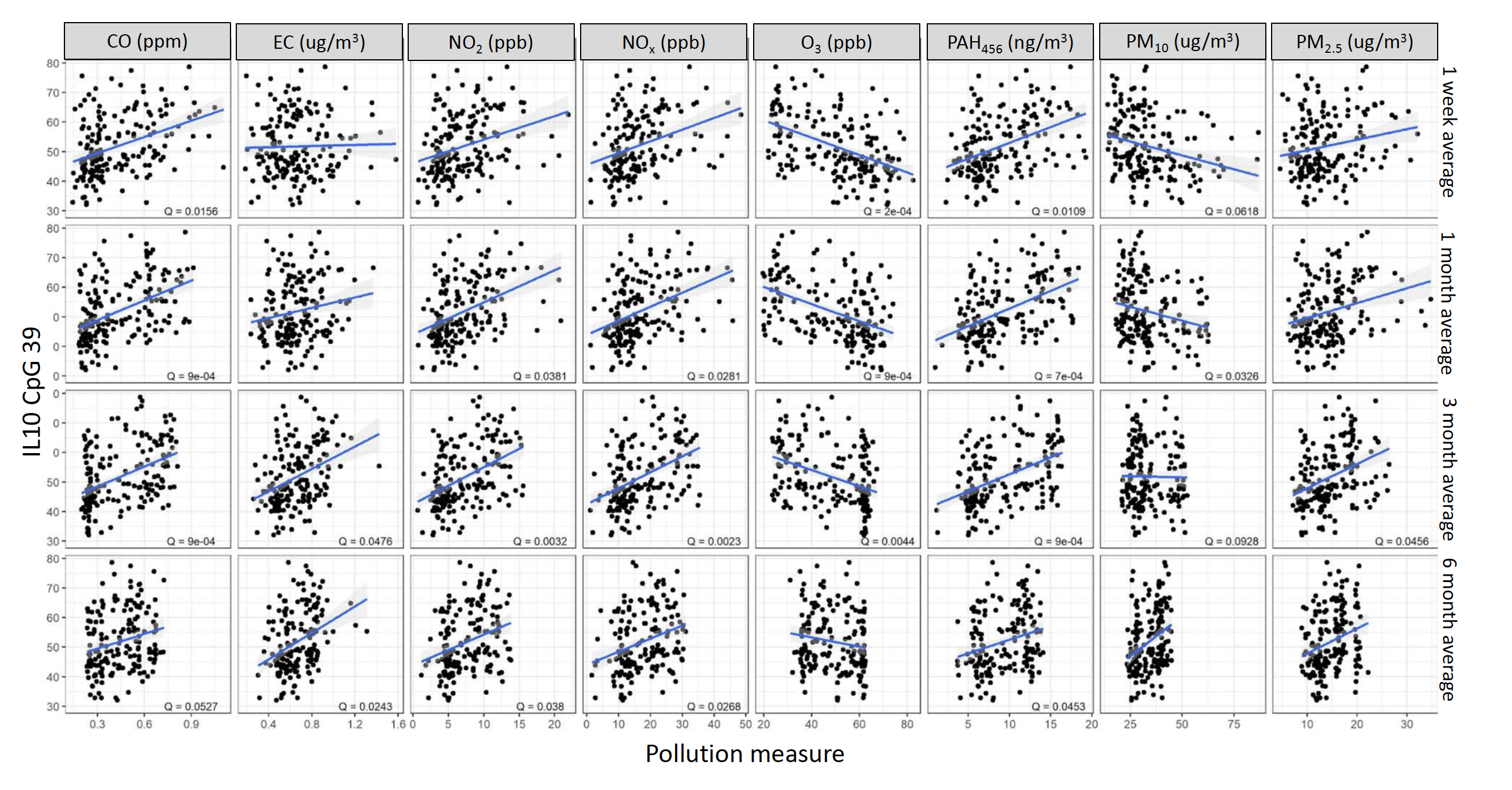

Supplement: Supplementary file 12 — Additional file 12: Figure S12. Associations between IL10_CpG39 site and Ambient Air Pollutant levels. Q value is the false-discovery-rate-adjusted p-value across all cell types, based on linear regression model adjusting for weight, age, season, race, and asthma diagnosis. Q < 0.1 are shown. CO: Carbon monoxide, EC: Elemental carbon, NO2: Nitric dioxide, NOx: Nitric oxides, O3: Ozone, PAH: Polycyclic aromatic hydrocarbons, PM: particulate matter. [file 13148_2022_1254_MOESM12_ESM.png]

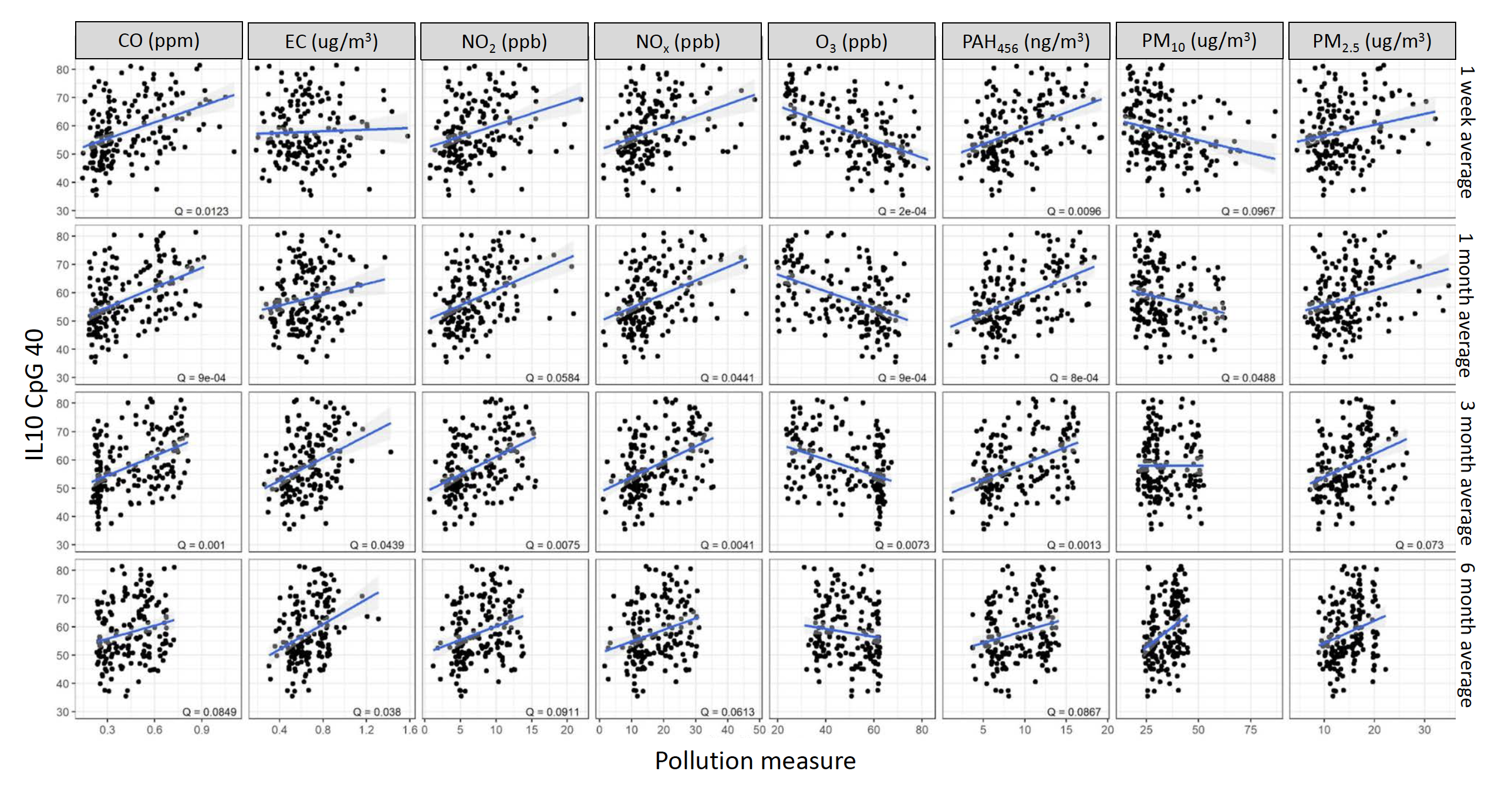

Supplement: Supplementary file 13 — Additional file 13: Figure S13. Associations between IL10_CpG40 site and Ambient Air Pollutant levels. Q value is the false-discovery-rate-adjusted p-value across all genes, based on linear regression model adjusting for weight, age, season, race, and asthma diagnosis. Q < 0.1 is considered statistically significant. CO: Carbon monoxide, EC: Elemental carbon, NO2: Nitric dioxide, NOx: Nitric oxides, O3: Ozone, PAH: Polycyclic aromatic hydrocarbons, PM: particulate matter. [file 13148_2022_1254_MOESM13_ESM.png]

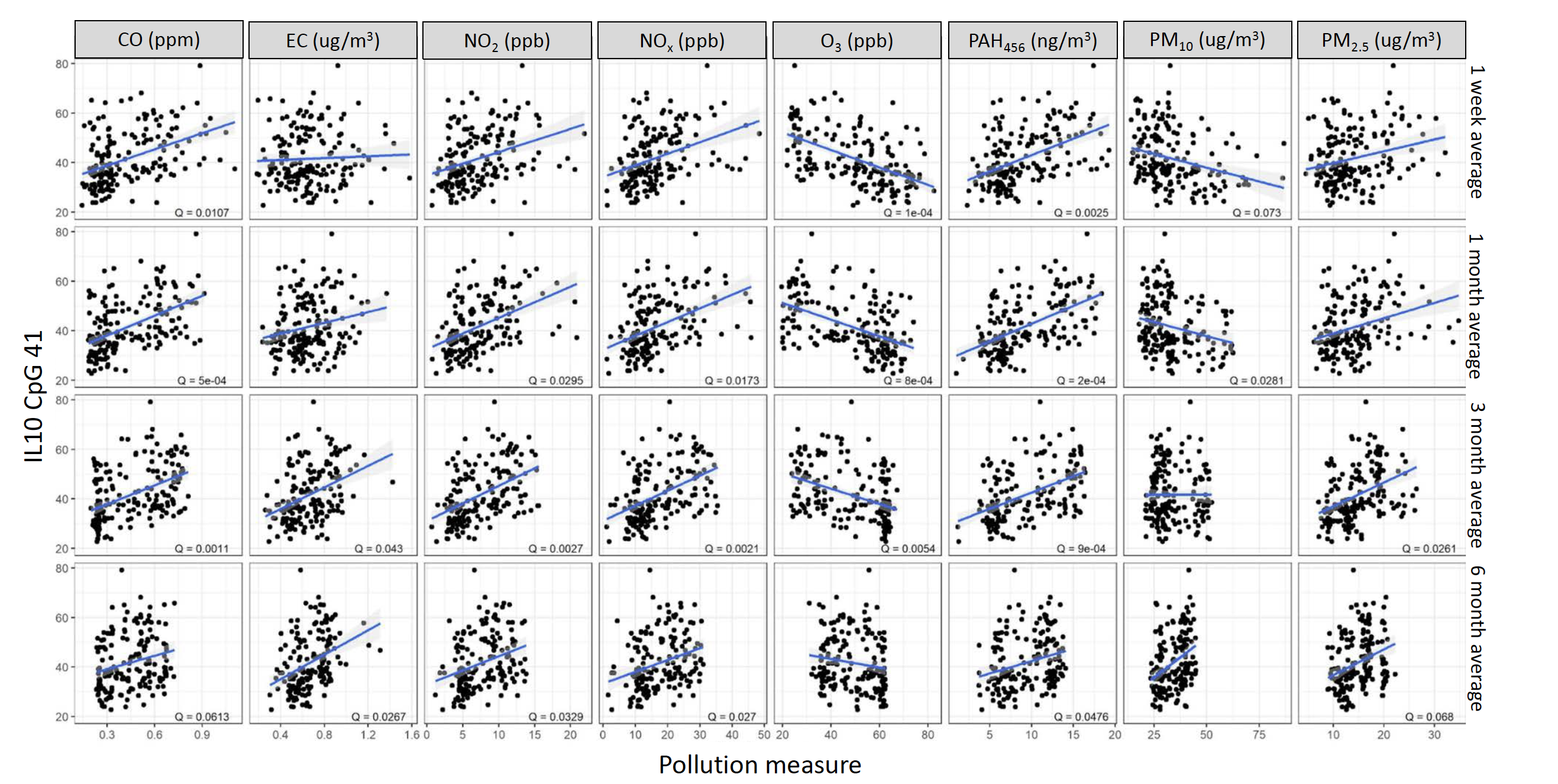

Supplement: Supplementary file 14 — Additional file 14: Figure S14. Associations between IL10_CpG41 site and Ambient Air Pollutant levels. Q value is the false-discovery-rate-adjusted p-value across all genes, based on linear regression model adjusting for weight, age, season, race, and asthma diagnosis. Q < 0.1 is considered statistically significant. CO: Carbon monoxide, EC: Elemental carbon, NO2: Nitric dioxide, NOx: Nitric oxides, O3: Ozone, PAH: Polycyclic aromatic hydrocarbons, PM: particulate matter. [file 13148_2022_1254_MOESM14_ESM.png]

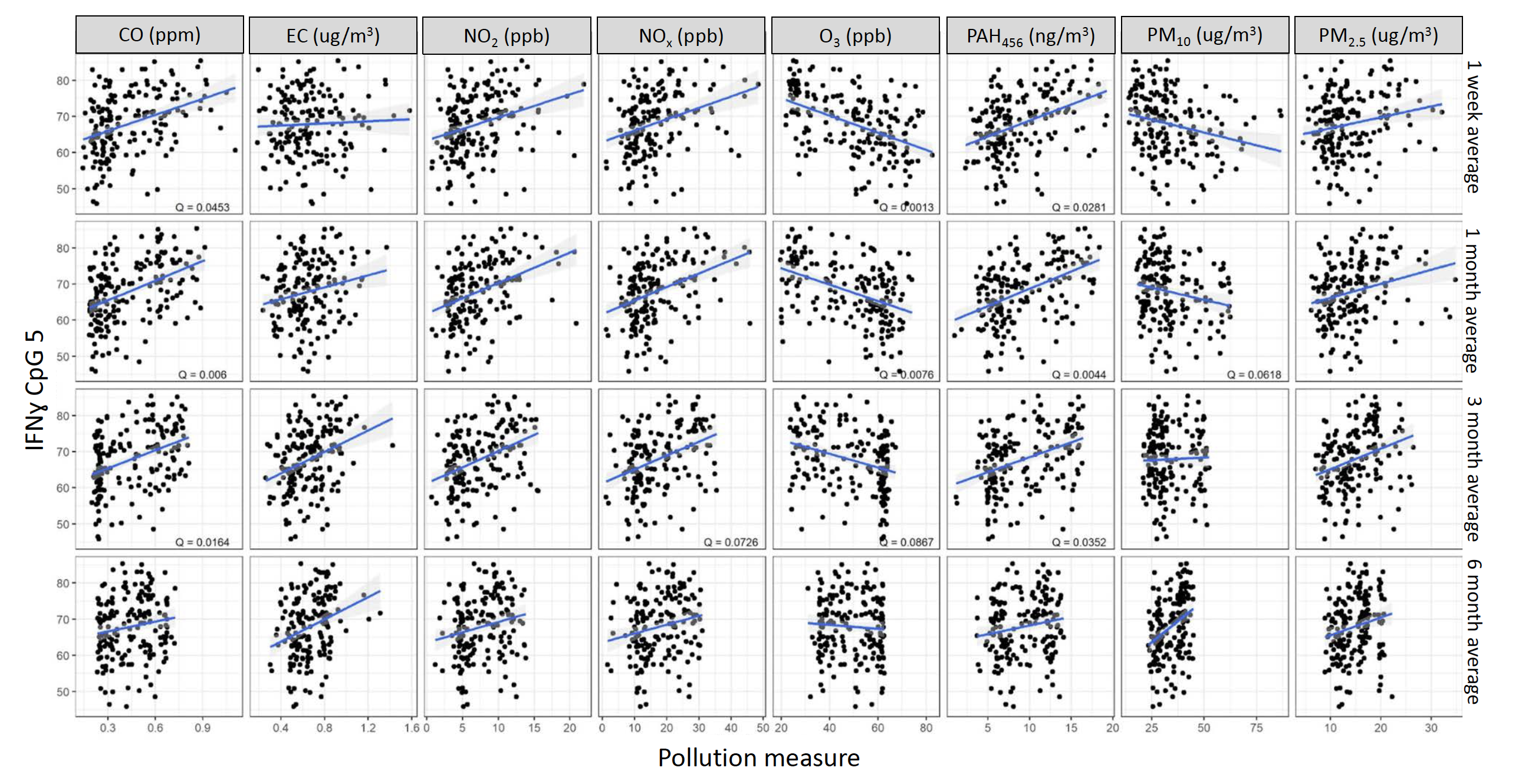

Supplement: Supplementary file 15 — Additional file 15: Figure S15. Associations between IFNγ_CpG5 site and Ambient Air Pollutant levels. Q value is the false-discovery-rate-adjusted p-value across all cell types, based on linear regression model adjusting for weight, age, season, race, and asthma diagnosis. Q < 0.1 are shown. CO: Carbon monoxide, EC: Elemental carbon, NO2: Nitric dioxide, NOx: Nitric oxides, O3: Ozone, PAH: Polycyclic aromatic hydrocarbons, PM: particulate matter. [file 13148_2022_1254_MOESM15_ESM.png]

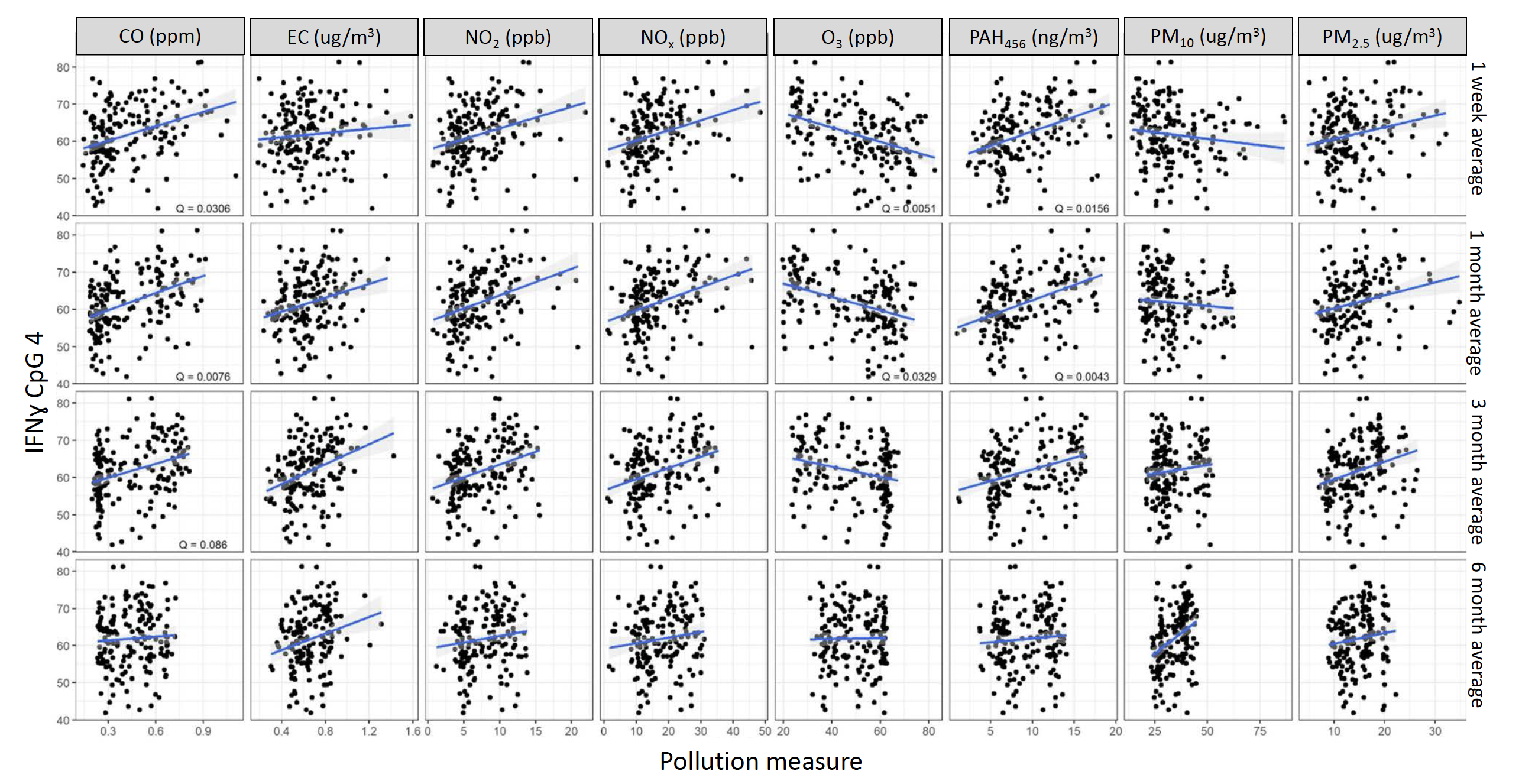

Supplement: Supplementary file 16 — Additional file 16: Figure S16. Associations between IFNγ_CpG4 site and Ambient Air Pollutant levels. Q value is the false-discovery-rate-adjusted p-value across all genes, based on linear regression model adjusting for weight, age, season, race, and asthma diagnosis. Q < 0.1 is considered statistically significant. CO: Carbon monoxide, EC: Elemental carbon, NO2: Nitric dioxide, NOx: Nitric oxides, O3: Ozone, PAH: Polycyclic aromatic hydrocarbons, PM: particulate matter. [file 13148_2022_1254_MOESM16_ESM.png]

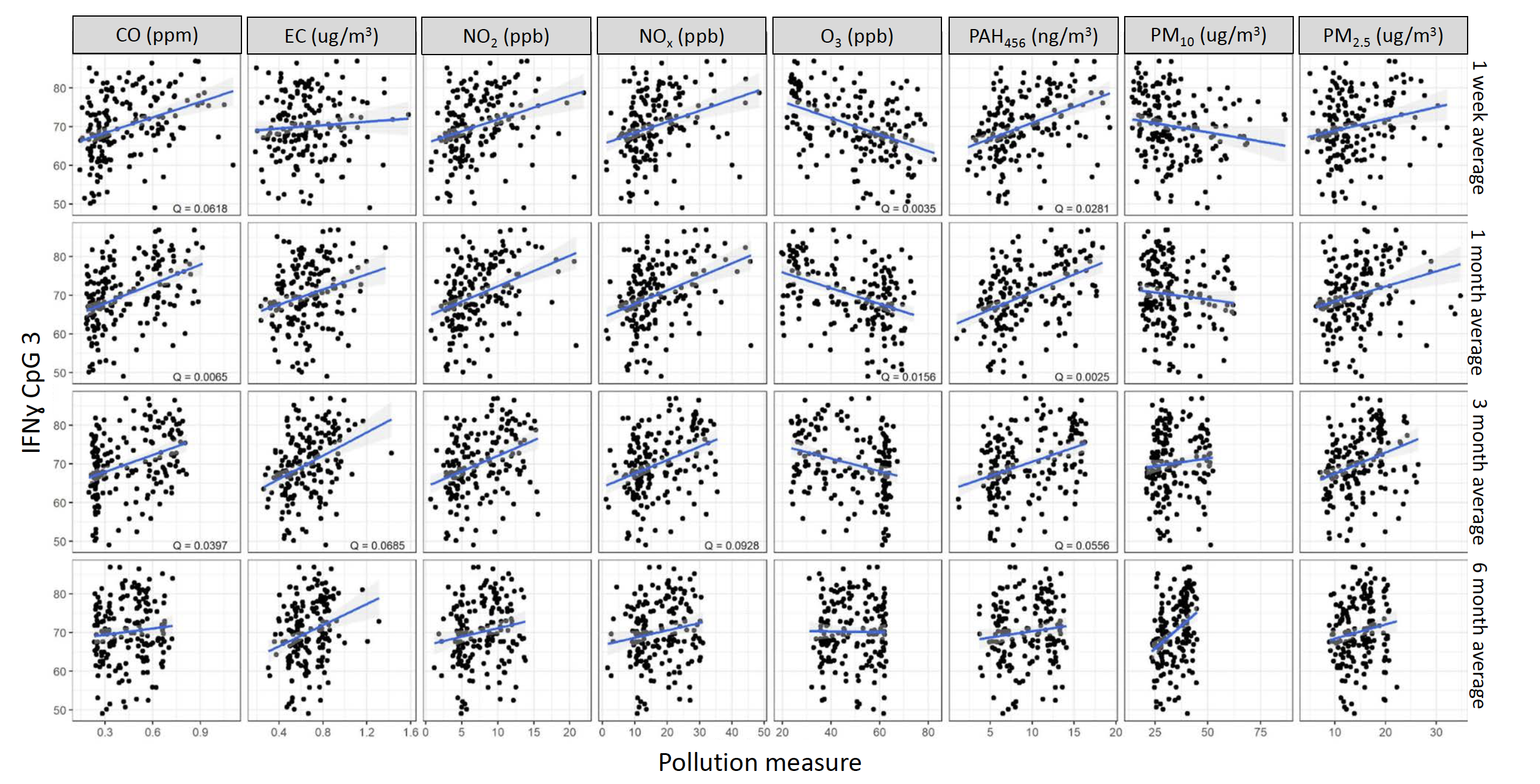

Supplement: Supplementary file 17 — Additional file 17: Figure S17. Associations between IFNγ_CpG3 site and Ambient Air Pollutant levels. Q value is the false-discovery-rate-adjusted p-value across all genes, based on linear regression model adjusting for weight, age, season, race, and asthma diagnosis. Q < 0.1 is considered statistically significant. CO: Carbon monoxide, EC: Elemental carbon, NO2: Nitric dioxide, NOx: Nitric oxides, O3: Ozone, PAH: Polycyclic aromatic hydrocarbons, PM: particulate matter. [file 13148_2022_1254_MOESM17_ESM.png]

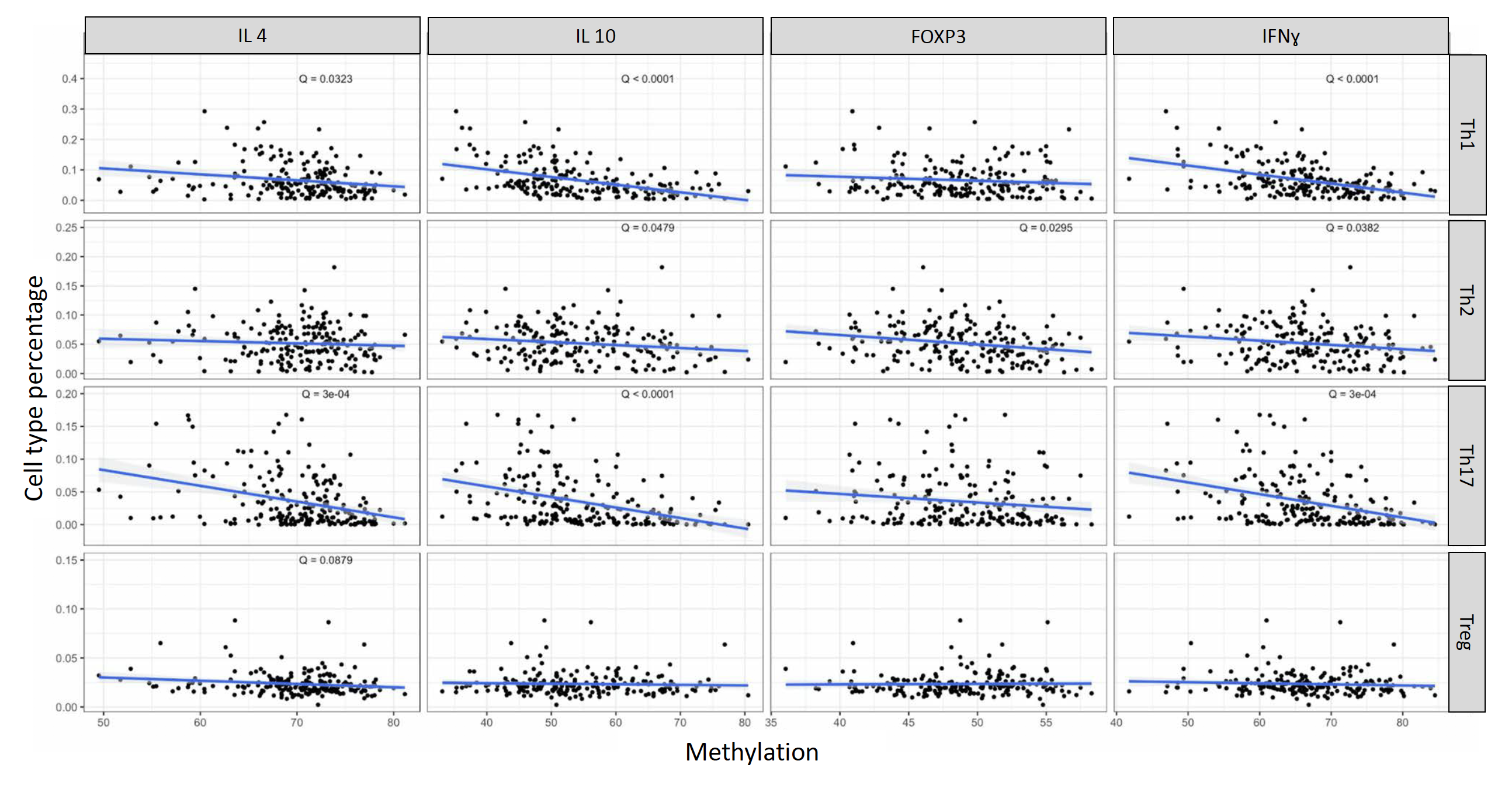

Supplement: Supplementary file 18 — Additional file 18: Figure S18. Associations between each cell type percentage vs each gene. Q value is the false-discovery-rate-adjusted p-value across all genes, based on linear regression model adjusting for weight, age, season, race, and asthma diagnosis. Q < 0.1 is considered statistically significant. [file 13148_2022_1254_MOESM18_ESM.png]
