# Supplementary material for: Increases in ambient air pollutants during pregnancy are linked to increases in methylation of IL4, IL10, and IFNγ
Source: Clin Epigenetics. 2022 Mar 14;14:40. doi: 10.1186/s13148-022-01254-2 (PMC8919561; doi:10.1186/s13148-022-01254-2)
Supplement: Supplementary file 19 — Additional file 19: Supplementary file with tables of (1) flow cytometry panel analysis, (2) summary of CpG site of the 4 genes Foxp3, Il-4, Il-10 and IFN-γ, (3) estimates and coefficients of gene and pollutant data by time estimate, and (4) estimates and correlation coefficients of mother and baby data. [file 13148_2022_1254_MOESM19_ESM.docx]

## Supplemental materials

**Supplemental Table 1: Flow cytometry panel analysis; 390 Samples/9 Colors**

| Flow cytometer antibodies | CD3, CD4, CD45RA, CD25, CD127, CXCR3, CCR4, CCR6 |
| --- | --- |
| Th1 | CD3+, CD4+, CXCR3+ |
| Th2 | CD3+, CD4+, CCR4+, CCR6- |
| Th17 | CD3+, CD4+, CCR4+, CCR6+ |
| Treg | CD3+, CD4+, CD25+, CD45RA-, CD127Low |

BioLegend flow cytometry antibodies: Zombie Green, CD3, CD4, CD25, CD45RA, CD127, CXCR3, CCR4, CCR6, Fluorochrome, FITC equivalent, Pacific Blue, PE, PE-Cy7, PerCP-Cy5.5, BV786, BV605, APC, BV650

**Supplemental Table 2:** Summary of CpG site of the 4 genes Foxp3, Il-4, Il-10 and IFN-γ

| **Gene** | **CpG site** | **Corresponding gene site** |
| --- | --- | --- |
| FoxP3 | 94, 95 | ChrX:49264916, ChrX:49264956 |
| IL4 | 3, 4, 21,  22, Loss 23, 24 | Chr5:132673938, Chr5:132673907, Chr5:132675095,  Chr5:132675115, Chr5:132675133, Chr5:132675242 |
| IL10 | 38, 39, 40, 41 | Chr1:206769266, Chr1:206769234, Chr1:206769230, Chr1:206769214 |
| IFNγ | 3, 4, 5 | Chr12:68159798, Chr12:68159930, Chr12:68160040 |

**Supplemental Table 3:Estimates and coefficients of gene and pollutant data by time estimate**

| Marker | Pollutant | Time | Coefficient (95% CI) | *p*-value | Q |
| --- | --- | --- | --- | --- | --- |
| **IL4** | **CO** | **1 week** | **5.48 (0.85, 10.1)** | **0.0214** | **0.0833** |
| **IL4** | **CO** | **1 month** | **7.38 (1.99, 12.77)** | **0.008** | **0.0466** |
| **IL4** | **CO** | **3 month** | **5.7 (0.89, 10.52)** | **0.0215** | **0.0833** |
| **IL4** | **CO** | **6 month** | **6.41 (0.76, 12.07)** | **0.0275** | **0.0928** |
| IL4 | PM_10_ | 1 week | -0.07 (-0.13, -0.01) | 0.0232 | 0.0874 |
| IL4 | PM_10_ | 1 month | -0.09 (-0.16, -0.03) | 0.0069 | 0.0442 |
| IL4 | PM_10_ | 3 month | -0.12 (-0.21, -0.03) | 0.0131 | 0.0569 |
| IL10 | CO | 1 week | 12.4 (4.95, 19.85) | 0.0013 | 0.0121 |
| IL10 | CO | 1 month | 19.26 (10.73, 27.79) | 0 | 7.00E-04 |
| IL10 | CO | 3 month | 16.13 (8.5, 23.75) | 1.00E-04 | 0.0013 |
| IL10 | CO | 6 month | 10.59 (1.29, 19.88) | 0.0268 | 0.0928 |
| IL10 | EC | 3 month | 10.8 (2.82, 18.78) | 0.0087 | 0.0467 |
| IL10 | EC | 6 month | 12.81 (3.83, 21.79) | 0.0058 | 0.0412 |
| IL10 | NO_2_ | 1 month | 0.63 (0.16, 1.1) | 0.0088 | 0.0467 |
| IL10 | NO_2_ | 3 month | 0.82 (0.37, 1.28) | 5.00E-04 | 0.0056 |
| IL10 | NO_2_ | 6 month | 0.57 (0.1, 1.03) | 0.0192 | 0.0792 |
| IL10 | NOx | 1 month | 0.28 (0.09, 0.47) | 0.005 | 0.0377 |
| IL10 | NOx | 3 month | 0.35 (0.17, 0.54) | 3.00E-04 | 0.0039 |
| IL10 | NOx | 6 month | 0.26 (0.06, 0.46) | 0.0118 | 0.056 |
| IL10 | O_3_ | 1 week | -0.28 (-0.39, -0.17) | 0 | 2.00E-04 |
| IL10 | O_3_ | 1 month | -0.23 (-0.33, -0.13) | 0 | 7.00E-04 |
| IL10 | O_3_ | 3 month | -0.17 (-0.27, -0.07) | 8.00E-04 | 0.0076 |
| IL10 | PAH_456_ | 1 week | 0.81 (0.36, 1.25) | 5.00E-04 | 0.0054 |
| IL10 | PAH_456_ | 1 month | 1.06 (0.62, 1.5) | 0 | 3.00E-04 |
| IL10 | PAH_456_ | 3 month | 0.81 (0.43, 1.2) | 1.00E-04 | 0.0013 |
| IL10 | PAH_456_ | 6 month | 0.54 (0.07, 1.01) | 0.0253 | 0.0924 |
| IL10 | PM_10_ | 1 week | -0.11 (-0.21, -0.01) | 0.0273 | 0.0928 |
| IL10 | PM_10_ | 1 month | -0.15 (-0.26, -0.04) | 0.0091 | 0.0467 |
| IL10 | PM_25_ | 3 month | 0.43 (0.09, 0.77) | 0.0132 | 0.0569 |
| FoxP3 | PM_10_ | 6 month | 0.18 (0.04, 0.32) | 0.0105 | 0.0514 |
| IFNγ | CO | 1 week | 8.5 (2.31, 14.69) | 0.0078 | 0.0466 |
| IFNγ | CO | 1 month | 13.05 (5.91, 20.19) | 4.00E-04 | 0.0054 |
| IFNγ | CO | 3 month | 9.09 (2.65, 15.53) | 0.0063 | 0.0423 |
| IFNγ | O_3_ | 1 week | -0.18 (-0.27, -0.09) | 1.00E-04 | 0.0022 |
| IFNγ | O_3_ | 1 month | -0.14 (-0.23, -0.05) | 0.0016 | 0.0136 |
| IFNγ | PAH_456_ | 1 week | 0.57 (0.2, 0.94) | 0.0028 | 0.0225 |
| IFNγ | PAH_456_ | month | 0.72 (0.35, 1.09) | 2.00E-04 | 0.0029 |
| IFNγ | PAH_456_ | 3 month | 0.42 (0.09, 0.75) | 0.0133 | 0.0569 |

Q value is the false-discovery-rate-adjusted p-value across all genes, based on linear regression model adjusting for weight, age, season, race, and asthma diagnosis.

**Supplemental Table 4: Estimates and correlation coefficients of mother and baby data**

| gene | group | Correlation coeffient (95% CI) | p.value | Q |
| --- | --- | --- | --- | --- |
| IL4 | cord | 0.3 (-0.06, 0.58) | 0.099 | 0.4794 |
| IL4 | year.1 | 0.05 (-0.24, 0.33) | 0.7338 | 0.9398 |
| IL4 | year.2 | 0.36 (-0.1, 0.69) | 0.1198 | 0.4794 |
| IL10 | cord | -0.05 (-0.4, 0.3) | 0.7698 | 0.9398 |
| IL10 | year.1 | 0.02 (-0.27, 0.31) | 0.8758 | 0.9398 |
| IL10 | year.2 | -0.03 (-0.47, 0.42) | 0.903 | 0.9398 |
| FoxP3 | cord | 0.16 (-0.2, 0.48) | 0.3743 | 0.8983 |
| FoxP3 | year.1 | -0.1 (-0.38, 0.19) | 0.4969 | 0.9398 |
| FoxP3 | year.2 | 0.57 (0.17, 0.81) | 0.0084 | 0.1012 |
| IFNγ | cord | 0.02 (-0.33, 0.37) | 0.9085 | 0.9398 |
| IFNγ | year.1 | -0.17 (-0.44, 0.12) | 0.2413 | 0.724 |
| IFNγ | year.2 | -0.02 (-0.47, 0.44) | 0.9398 | 0.9398 |

Q value is the false-discovery-rate-adjusted p-value across all genes, based on linear regression model adjusting for weight, age, season, race, and asthma diagnosis.

## Supplemental figure legends:

**Supplemental Figure 1. Ambient air pollutant (AAP) concentration levels and counts per participant.** CO: Carbon monoxide, EC: Elemental carbon, NO2: Nitric dioxide, NOx: Nitric oxides, O3: Ozone, PAH: Polycyclic aromatic hydrocarbons, PM: particulate matter.

**Supplemental Figure 2.** **Associations between Foxp3 and Ambient Air Pollutant levels**. Q value is the false-discovery-rate-adjusted p-value across all genes, based on linear regression model adjusting for weight, age, season, race, and asthma diagnosis. Q<0.1 is considered statistically significant. FoxP3 refers to average DNA methylation across 3 CpG sites in the gene. CO: Carbon monoxide, EC: Elemental carbon, NO2: Nitric dioxide, NOx: Nitric oxides, O3: Ozone, PAH: Polycyclic aromatic hydrocarbons, PM: particulate matter.

**Supplemental Figure 3.** **Associations between Treg percentage and Ambient Air Pollutant levels.** Q value is the false-discovery-rate-adjusted p-value across all genes, based on linear regression model adjusting for weight, age, season, race, and asthma diagnosis. Q<0.1 is considered statistically significant. CO: Carbon monoxide, EC: Elemental carbon, NO2: Nitric dioxide, NOx: Nitric oxides, O3: Ozone, PAH: Polycyclic aromatic hydrocarbons, PM: particulate matter.

**Supplemental Figure 4.** **Associations between each T cell sublet percentage and each CpG site methylation.** Q value is the false-discovery-rate-adjusted p-value across all genes, based on linear regression model adjusting for weight, age, season, race, and asthma diagnosis. Q<0.1 is considered statistically significant.

**Supplemental Figure 5.** **Associations between IL4_CpG4 site and Ambient Air Pollutant levels.** Q value is the false-discovery-rate-adjusted p-value across all genes, based on linear regression model adjusting for weight, age, season, race, and asthma diagnosis. Q<0.1 is considered statistically significant. CO: Carbon monoxide, EC: Elemental carbon, NO2: Nitric dioxide, NOx: Nitric oxides, O3: Ozone, PAH: Polycyclic aromatic hydrocarbons, PM: particulate matter.

**Supplemental Figure 6.** **Associations between IL4_CpG3 site and Ambient Air Pollutant levels.** Q value is the false-discovery-rate-adjusted p-value across all genes, based on linear regression model adjusting for weight, age, season, race, and asthma diagnosis. Q<0.1 is considered statistically significant. CO: Carbon monoxide, EC: Elemental carbon, NO2: Nitric dioxide, NOx: Nitric oxides, O3: Ozone, PAH: Polycyclic aromatic hydrocarbons, PM: particulate matter.

**Supplemental Figure 7.** **Associations between IL4_CpG24 site and Ambient Air Pollutant levels.** Q value is the false-discovery-rate-adjusted p-value across all genes, based on linear regression model adjusting for weight, age, season, race, and asthma diagnosis. Q<0.1 is considered statistically significant. CO: Carbon monoxide, EC: Elemental carbon, NO2: Nitric dioxide, NOx: Nitric oxides, O3: Ozone, PAH: Polycyclic aromatic hydrocarbons, PM: particulate matter.

**Supplemental Figure 8.** **Associations between IL4_CpG22 site and Ambient Air Pollutant levels.** Q value is the false-discovery-rate-adjusted p-value across all genes, based on linear regression model adjusting for weight, age, season, race, and asthma diagnosis. Q<0.1 is considered statistically significant. CO: Carbon monoxide, EC: Elemental carbon, NO2: Nitric dioxide, NOx: Nitric oxides, O3: Ozone, PAH: Polycyclic aromatic hydrocarbons, PM: particulate matter.

**Supplemental Figure 9.** **Associations between IL4_CpG21 site and Ambient Air Pollutant levels.** Q value is the false-discovery-rate-adjusted p-value across all genes, based on linear regression model adjusting for weight, age, season, race, and asthma diagnosis. Q<0.1 is considered statistically significant. CO: Carbon monoxide, EC: Elemental carbon, NO2: Nitric dioxide, NOx: Nitric oxides, O3: Ozone, PAH: Polycyclic aromatic hydrocarbons, PM: particulate matter.

**Supplemental Figure 10.** **Associations between IL4_lossCpG23 site and Ambient Air Pollutant levels.** Q value is the false-discovery-rate-adjusted p-value across all genes, based on linear regression model adjusting for weight, age, season, race, and asthma diagnosis. Q<0.1 is considered statistically significant. CO: Carbon monoxide, EC: Elemental carbon, NO2: Nitric dioxide, NOx: Nitric oxides, O3: Ozone, PAH: Polycyclic aromatic hydrocarbons, PM: particulate matter.

**Supplemental Figure 11.** **Associations between IL10_CpG38 site and Ambient Air Pollutant levels.** Q value is the false-discovery-rate-adjusted p-value across all genes, based on linear regression model adjusting for weight, age, season, race, and asthma diagnosis. Q<0.1 is considered statistically significant. CO: Carbon monoxide, EC: Elemental carbon, NO2: Nitric dioxide, NOx: Nitric oxides, O3: Ozone, PAH: Polycyclic aromatic hydrocarbons, PM: particulate matter.

**Supplemental Figure 12.** **Associations between IL10_CpG39 site and Ambient Air Pollutant levels.** Q value is the false-discovery-rate-adjusted p-value across all cell types, based on linear regression model adjusting for weight, age, season, race, and asthma diagnosis. Q<0.1 are shown. CO: Carbon monoxide, EC: Elemental carbon, NO2: Nitric dioxide, NOx: Nitric oxides, O3: Ozone, PAH: Polycyclic aromatic hydrocarbons, PM: particulate matter.

**Supplemental Figure 13.** **Associations between IL10_CpG40 site and Ambient Air Pollutant levels.** Q value is the false-discovery-rate-adjusted p-value across all genes, based on linear regression model adjusting for weight, age, season, race, and asthma diagnosis. Q<0.1 is considered statistically significant. CO: Carbon monoxide, EC: Elemental carbon, NO2: Nitric dioxide, NOx: Nitric oxides, O3: Ozone, PAH: Polycyclic aromatic hydrocarbons, PM: particulate matter.

**Supplemental Figure 14.** **Associations between IL10_CpG41 site and Ambient Air Pollutant levels.** Q value is the false-discovery-rate-adjusted p-value across all genes, based on linear regression model adjusting for weight, age, season, race, and asthma diagnosis. Q<0.1 is considered statistically significant. CO: Carbon monoxide, EC: Elemental carbon, NO2: Nitric dioxide, NOx: Nitric oxides, O3: Ozone, PAH: Polycyclic aromatic hydrocarbons, PM: particulate matter.

**Supplemental Figure 15.** **Associations between IFNγ_CpG5 site and Ambient Air Pollutant levels.** Q value is the false-discovery-rate-adjusted p-value across all cell types, based on linear regression model adjusting for weight, age, season, race, and asthma diagnosis. Q<0.1 are shown. CO: Carbon monoxide, EC: Elemental carbon, NO2: Nitric dioxide, NOx: Nitric oxides, O3: Ozone, PAH: Polycyclic aromatic hydrocarbons, PM: particulate matter.

**Supplemental Figure 16.** **Associations between IFNγ_CpG4 site and Ambient Air Pollutant levels.** Q value is the false-discovery-rate-adjusted p-value across all genes, based on linear regression model adjusting for weight, age, season, race, and asthma diagnosis. Q<0.1 is considered statistically significant. CO: Carbon monoxide, EC: Elemental carbon, NO2: Nitric dioxide, NOx: Nitric oxides, O3: Ozone, PAH: Polycyclic aromatic hydrocarbons, PM: particulate matter.

**Supplemental Figure 17.** **Associations between IFNγ_CpG3 site and Ambient Air Pollutant levels.** Q value is the false-discovery-rate-adjusted p-value across all genes, based on linear regression model adjusting for weight, age, season, race, and asthma diagnosis. Q<0.1 is considered statistically significant. CO: Carbon monoxide, EC: Elemental carbon, NO2: Nitric dioxide, NOx: Nitric oxides, O3: Ozone, PAH: Polycyclic aromatic hydrocarbons, PM: particulate matter.

**Supplemental Figure 18.** **Associations between each cell type percentage vs each gene**. Q value is the false-discovery-rate-adjusted p-value across all genes, based on linear regression model adjusting for weight, age, season, race, and asthma diagnosis. Q<0.1 is considered statistically significant.
